# TopologyNet: Topology based deep convolutional and multi-task neural networks for biomolecular property predictions

**DOI:** 10.1371/journal.pcbi.1005690

**Published:** 2017-07-27

**Authors:** Zixuan Cang, Guo-Wei Wei

**Affiliations:** 1 Department of Mathematics, Michigan State University, East Lansing, MI 48824, USA; 2 Department of Biochemistry and Molecular Biology, Michigan State University, East Lansing, MI 48824, USA; 3 Department of Electrical and Computer Engineering, Michigan State University, East Lansing, MI 48824, USA; Fox Chase Cancer Center, UNITED STATES

## Abstract

Although deep learning approaches have had tremendous success in image, video and audio processing, computer vision, and speech recognition, their applications to three-dimensional (3D) biomolecular structural data sets have been hindered by the geometric and biological complexity. To address this problem we introduce the element-specific persistent homology (ESPH) method. ESPH represents 3D complex geometry by one-dimensional (1D) topological invariants and retains important biological information via a multichannel image-like representation. This representation reveals hidden structure-function relationships in biomolecules. We further integrate ESPH and deep convolutional neural networks to construct a multichannel topological neural network (TopologyNet) for the predictions of protein-ligand binding affinities and protein stability changes upon mutation. To overcome the deep learning limitations from small and noisy training sets, we propose a multi-task multichannel topological convolutional neural network (MM-TCNN). We demonstrate that TopologyNet outperforms the latest methods in the prediction of protein-ligand binding affinities, mutation induced globular protein folding free energy changes, and mutation induced membrane protein folding free energy changes. Availability: weilab.math.msu.edu/TDL/

## Author summary

The predictions of biomolecular functions and properties from biomolecular structures are of fundamental importance in computational biophysics. The structural and biological complexities of biomolecules and their interactions hinder successful predictions. Machine learning has become an important tool for such predictions. Recent advances in deep learning architectures, particularly convolutional neural network (CNN), have profoundly impacted a number of disciplines, such as image classification and voice recognition. Though CNN can be directly applied to molecular sciences by using a three-dimensional (3D) image-like brute-force representation, it is computationally intractable when applied to large biomolecules and large datasets. We propose a topological strategy to significantly reduce the structural and biological complexity of biomolecules and provide an efficient topology based CNN architecture. Element-specific persistent homology, a new algebraic topology, has been developed to cast biomolecules in a multichannel image-like representation suitable for CNN. The power of the proposed topology based neural network (TopologyNet) is further enhanced by auxiliary descriptors and a multi-task deep learning architecture. It has been demonstrated that TopologyNet framework outperforms other methods in the predictions of protein-ligand binding affinities and mutation induced protein stability changes.

This is a *PLOS Computational Biology* Methods paper.

## Introduction

Understanding the structure-function relationships of biomolecules is fundamentally important in computational biophysics and experimental biology. As such, methods that can robustly predict biomolecular properties, such as protein-ligand binding affinity and protein stability change upon mutation from three-dimensional (3D) structures are important tools to help us understand this relationship. Numerous approaches have been developed to unveil the structure-function relationship. Physics based models make use of fundamental laws of physics, i.e., quantum mechanics, molecular mechanics, continuum mechanics, multiscale modeling, statistical mechanics, thermodynamics, etc, to investigate structure-function relationships and predict function from structure. Physical methods provide important insights and are indispensable for understanding the relationships between protein structure and function.

The exponential growth of biological data has set the stage for data-driven discovery of structure-function relationships. Indeed, the Protein Data Bank (PDB) has accumulated near 130,000 tertiary structures. The availability of 3D structural data enables knowledge based approaches to offer complementary and competitive predictions of structure-function relationships. Recent advances in machine learning algorithms have made data driven approaches more competitive and powerful than ever. Arguably, machine learning is one of the most important developments in data analysis. Machine learning has become an indispensable tool in biomolecular data analysis and prediction. Virtually every computational problem in computational biology and biophysics, such as the prediction of solvation free energies, protein-ligand binding affinities, mutation impacts, pKa values, etc, has a class of knowledge based approaches that are either parallel or complementary to physics based approaches. The ability to recognize nonlinear and high-order interactions among features as well as the capability of handling data with underlying spatial dimensions hierarchically has lead to breakthroughs in deep convolutional neural networks in image processing, video, audio and computer vision [1, 2]. Likewise, recurrent nets have shed light on sequential data such as text and speech [3, 4]. Deep learning has fueled the rapid growth in several areas of data science [3, 4]. Machine learning based approaches are advantageous due to their ability to handle large data sets and nonlinear relationships in physically derived descriptors. Notably, deep learning can automatically extract optimal high level features and discover intricate structures in large data sets.

Given multiple learning tasks, multi-task learning (MTL) [5] provides a powerful tool to exploit the intrinsic relatedness among learning tasks, transfer predictive information among tasks, and achieve better generalized performance than single task learning. During the learning stage, MTL algorithms seek to learn a shared representation (e.g., shared distribution of a given hyper-parameter [6], shared low-rank subspace [7], shared feature subset [8] and clustered task structure [9]), and use the shared representation to bridge between tasks and transfer knowledge. MTL has applications to the bioactivity of small molecular drugs [10–12] and genomics [13]. Linear regression based MTL heavily depends on well crafted features, while neural network based MTL allows more flexible task coupling and is able to deliver satisfactory results with a large number of low level features provided such features have the representative power of the problem.

For complex 3D biomolecular data, the physical features used in machine learning vary greatly in their nature. Typical features are generated from geometric properties, electrostatics, atom types, atomic partial charges, and graph theory based properties [14]. Such manually extracted features can be used in a deep neural network, but the performance heavily relies on feature engineering. In contrast, convolutional neural networks learn to extract high level representations hierarchically from low level features while maintaining the underlying spatial relationships. However, the cost is huge for directly applying convolutional neural network to the 3D biomolecules, especially if long-range interactions are included. A major obstacle in the development of deep learning nets for 3D biomolecular data is their entanglement between geometric complexity and biological complexity.

Most theoretical models for the study of structure-function relationships of biomolecules are based on geometric modeling techniques. Mathematically, these approaches exploit local geometric information, i.e., coordinates, distances, angles, areas, and sometimes curvatures [15] for the physical modeling of biomolecular systems. Indeed, the importance of geometric modeling for structural biology [16], and biophysics cannot be overemphasized. However, geometry based models often contain too much structural detail and are frequently computationally intractable for large structures or datasets. In many biological problems, such as the opening or closing of ion channels, the association or dissociation of binding ligands, the folding or unfolding of proteins, and the symmetry breaking or formation of virus capsids, obvious topological changes exist. In fact, one only needs qualitative topological information to understand many physical and biological functions. In short, *topology-function relationships* exist in many biomolecular systems.

Topology offers entirely different approaches and could provide significant simplification of biomolecular data [17–24]. The study of topology deals with the connectivity of different components in a space, and characterizes independent entities, rings and higher dimensional faces within the space [25]. Topological methods produce a high level of abstraction to many biological processes. For example, the opening and closing of ion channels, the assembly or disassembly of virus capsids, the folding and unfolding of proteins, and the association or dissociation of ligands are reflected by topological changes. The fundamental task of topological data analysis is to extract topological invariants, namely the intrinsic features of the underlying space, of a given data set without additional structure information. Examples include covalent bonds, hydrogen bonds, van der Waals interactions, etc. A fundamental concept in algebraic topology is simplicial homology, which concerns the identification of topological invariants from a set of discrete node coordinates such as atomic coordinates in a protein or a protein-ligand complex. For a given (protein) configuration, number of independent components, rings and cavities are topological invariants and they are refered to as Betti-0, Betti-1 and Betti-2 numbers respectively. Conventional topology or homology is truly free of metrics or coordinates, and thus retains too little geometric information to be practically useful for the predictions of biomolecular properties. To address this issue, spatial scales are embeded in the topology, which equips the topological representations with geometric information.

Persistent homology is a relatively new branch of algebraic topology that embeds multiscale geometric information in topological invariants to achieve an interplay between geometry and topology. It creates a variety of topologies of a given object by varying a filtration parameter, such as the radii of balls centered at the nodes or the level set of a surface function. As a result, persistent homology can capture topological structures continuously over a range of spatial scales. Unlike commonly used computational homology which results in truly metric free representations, persistent homology embeds geometric information in topological invariants, e.g., Betti numbers so that “birth” and “death” of isolated components, circles, rings, voids or cavities can be monitored at any geometric scale by topological measurements. In the past decade, persistent homology has been developed as a new multiscale representation of topological features. The 0-th dimensional version was originally introduced for computer vision applications under the name “size function” [26, 27]. Persistent homology theory and subsequent algorithms were formulated by Edelsbrunner et al. [28]. Later, a more general theory was developed by Zomorodian and Carlsson [18]. Since that time, there have been significant theoretical development [29–37] as well as various computational algorithms [38–43]. Persistent homology is often visualized by the use of barcodes [44, 45], where horizontal line segments or bars represent homology generators that survive over different filtration scales.

Persistent homology has been applied to computational biology [46–48], in the mathematical modeling and prediction of nano particles, proteins and other biomolecules [47, 49, 50]. Previously, we have introduced molecular topological fingerprint (TF) to reveal topology-function relationships in protein folding and protein flexibility [49]. Contrary to many other fields where short-lived topological events are considered noise, we have shown that such short-lived properties are in fact important components in biomolecular analysis and should be included in molecular topological fingerprints. Quantitative topological analysis has been cultivated to predict the curvature energy of fullerene isomers [50, 51] and protein folding stability [49]. Differential geometry based persistent homology [51], multidimensional persistence [52], and multiresolutional persistent homology [53, 54] have been proposed to better characterize biomolecular data [52], detect protein cavities [55], and resolve ill-posed inverse problems in cryo-EM structure determination [56]. A persistent homology based machine learning algorithm has also been developed for protein structural classification [57]. However, ordinary persistent homology oversimplifies biological information. Consequently, persistent homology based machine learning algorithms are not as competitive as other conventional techniques in protein structural classification [57, 58].

The objective of the present work is to introduce a new framework for the structure based biomolecular property predictions using element-specific persistent homology, and convolutional and multi-task neural networks. In this framework, element-specific persistent homology reduces geometric and biological complexities and provides a sufficient and structured low level representation for neural networks. Given this representation, convolutional neural networks can then learn from data to extract high level representations of the biomolecular systems, while retaining the spatial relationships, and construct mappings from these representations to the target properties. For the prediction problems whose available datasets are small, multi-task learning by jointly learning the related prediction problems with larger available datasets helps to extract a proper high level representation for the target applications. The element-specific treatment is inspired by the RF-score method [59] for binding affinity prediction. Element-specific persistent homology is originated in our previous work using classic machine learning methods. [60, 61] In this work, we further develop topology based neural network (TopologyNet) models for the predictions of biomolecular structure-function relationships. Specifically, we integrate ESPH and convolutional neural networks (CNNs) to improve modern methods for protein-ligand binding affinity and protein mutation impact predictions from 3D biomolecular data. In this approach, topological invariants are used to reduce the dimensionality of 3D biomolecular data. Additionally, element-specific persistent barcodes offer image-like topological representations to facilitate convolutional deep neural networks. Moreover, biological information is retained by element-specific topological fingerprints and described in multichannels in our image like representation. Furthermore, convolutional neural networks uncover hidden relationships between biomolecular topological invariants and biological functions. Finally, a multi-task multichannel topological convolutional neural network (MM-TCNN) framework is introduced to exploit the relations among various structure-function predictions and enhance the prediction for problems with small and noisy training data. Our hypothesis is that many biomolecular predictions share a common set of topological fingerprints representations and are highly correlated to each other. As a result, multi-task deep learning by simultaneous training for globular proteins and membrane proteins improves upon existing predictions for the mutation induced stability changes of membrane proteins whose training data size is relatively small.

## Results

### Deep learning prediction of protein-ligand binding affinities

Protein-ligand binding is a fundamental biological process in cells and involves detailed molecular recognition, synergistic protein-ligand interaction, and may involve protein conformational changes. Agonist binding is crucial to receptor functions and typically triggers a physiological response, such as transmitter-mediated signal transduction, hormone and growth factor regulated metabolic pathways, stimulus-initiated gene expression, enzyme production, cell secretion, etc. Understanding protein-ligand interactions has been a fundamental issue in molecular biophysics, structural biology and medicine. A specific task in drug and protein design is to predict protein-ligand binding affinity from given structural information [62] Protein-ligand binding affinity is a measurement of rate of binding which indicates the degree of occupancy of a ligand at the corresponding protein binding site and is affected by several factors including intermolecular interaction strength and solvation effects. The ability to predict protein-ligand binding affinity to a desired accuracy is a prerequisite for the success of many applications in biochemistry such as protein-ligand docking and drug discovery. In general, there are three types of binding affinity predictors (commonly called scoring functions): physics based [63, 64], empirical [65–72], and knowledge based [73–75]. In general, physics based scoring functions invoke QM and QM/MM approaches [76, 77] to provide unique insights into the molecular mechanism of protein-ligand interactions. A prevalent view is that binding involves intermolecular forces, such as steric contacts, ionic bonds, hydrogen bonds, hydrophobic effects and van der Waals interactions. Empirical scoring functions work well but require carefully selected data sets and parametrization [65–68]. However, both physics based scoring functions and empirical scoring functions employ linear superposition principles that are not explicitly designed to deal with exponentially growing and increasingly diverse experimental data sets. Knowledge based scoring functions use modern machine learning techniques, which utilize nonlinear regression and exploit large data sets to uncover underlying patterns within the data sets. Given the current massive and complex data challenges, knowledge based scoring functions outperform other scoring functions. [65].

In this study, the proposed method is tested on the PDBBind 2007 data set [78]. The PDBBind 2007 core set of 195 protein-ligand complexes is used as the test set and the PDBBind 2007 refined set, excluding the PDBBind 2007 core set, is used as the training set with 1105 protein-ligand complexes. A comparison between our TNet-binding predictor (TNet-BP) and other binding affinity predictors is summarized in Table 1. TNet-BP outperforms all the other scoring functions reported by Li *et al* [59] on the task of binding affinity prediction from structures.

**Table 1:** Performance comparisons of TNet-BP and other methods.

Method	*R*_*P*_	RMSE
TNet-BP	0.826^a^	1.37
RF::VinaElem	0.803	1.42
RF:Vina	0.739	1.61
Cyscore	0.660	1.79
X-Score::HMScore	0.644	1.83
MLR::Vina	0.622	1.87
HYDE2.0::HbondsHydrophobic	0.620	1.89
DrugScore	0.569	1.96
SYBYL::ChemScore	0.555	1.98
AutoDock Vina	0.554	1.99
DS::PLP1	0.545	2.00
GOLD::ASP	0.534	2.02
SYBYL::G-Score	0.492	2.08
DS::LUDI3	0.487	2.09
DS:LigScore2	0.464	2.12
GlideScore-XP	0.457	2.14
DS::PMF	0.445	2.14
GOLD::ChemScore	0.441	2.15
PHOENIX	0.616	2.16
SYBYL::D-Score	0.392	2.19
DS::Jain	0.316	2.24
IMP::RankScore	0.322	2.25
GOLD::GoldScore	0.295	2.29
SYBYL::PMF-Score	0.268	2.29
SYBYL::F-Score	0.216	2.35

Comparison of optimal Pearson correlation coefficients *R*_*P*_ and RMSEs (*pK*_*d*_/*pK*_*i*_) of various scoring functions for the prediction of protein-ligand binding affinity of the PDBBind 2007 core set. Except for the result of our TNet-BP, all other results are adopted from Li *et al* [59].

*^a^* Median results (The best *R*_*P*_ = 0.828 and best RMSE = 1.37 for this method).

TNet-BP is also validated on a larger dataset, PDBBind v2016 refined set of 4057 complexes, where the training set contains 3767 samples which is the refined set minus the core set, and the testing set is the core set with 290 samples. All the model parameters and training procedures are the same as that used for v2007 dataset except that the epoch number is set to 500 instead of 2000 due to the larger data size. The median *R*_*P*_ and RMSE are 0.81 and 1.34 pKd/pKi units, respectively.

### Deep learning prediction of protein folding free energy changes upon mutation

Apart from some unusual exceptions, proteins fold into specific three-dimensional structures to provide the structural basis for living organisms. Protein functions, i.e., acting as enzymes, cell signaling mediators, ligand receptors, and structural supports, are typical consequences of a delicate balance between protein structural stability and flexibility. Mutation that changes protein amino acid sequences through non-synonymous single nucleotide substitutions (nsSNPs) plays a fundamental role in selective evolution. Such substitutions may lead to the loss or the modification of certain functions. Mutations are often associated with various human diseases [79, 80]. For example, mutations in proteases and their natural inhibitors result in more than 60 human hereditary diseases [81]. Additionally, mutation can also lead to drug resistance [82]. Artificially designed mutations are used to understand mutation impacts to protein structural stability, flexibility and function, as well as mutagenic diseases, and evolution pathways of organisms [83]. However, mutagenesis experiments are typically costly and time-consuming. Computational prediction of mutation impacts is able to systematically explore protein structural instabilities, functions, disease connections, and organismal evolution pathways [84] and provide an economical, fast, and potentially accurate alternative to mutagenesis experiments. Many computational methods have been developed in the past decade, including support vector machine based approach [85], statistical potentials based approach [86], knowledge-modified MM/PBSA approach [87], Rosetta protocols [88], FoldX (3.0, beta 6.1) [84], SDM [89], DUET [90], PPSC (Prediction of Protein Stability, version 1.0) with the 8 (M8) and 47 (M47) feature sets [91], PROVEAN [92], ELASPIC [93], STRUM [94], and EASE-MM [95].

The proposed method is tested on a data set of 2648 mutation instances of 131 proteins named “S2648” data set [86] in a 5-fold cross validation task over the “S2648” set and a task of prediction of the “S350” set which is a subset of “S2648” set. The “S2648” set, excluding the “S350” subset, is used as the training set in the prediction of the “S350” set. All thermodynamic data are obtained from the ProTherm database [96]. A comparison of the performance of various methods is summarized in Table 2. Among them, STRUM [94] is based on structural, evolutionary and sequence information and results in excellent performance. We therefore have constructed two topology based neural network mutation predictors (TNet-MPs). TNet-MP-1 is solely based on topological information while TNet-MP-2 is aided by auxiliary features characterizing electrostatics, evolutionary, and sequence information, which is merged into the convolutional neural network at one of the fully connected layers. TNet-MP-2 is able to significantly improve our original topological prediction, indicating the importance of the aforementioned auxiliary information to mutation prediction. The details of handcrafted features can be found in S1 Text. Handcrafted features.

**Table 2:** Performance comparisons of TNet-MP and other methods.

Method	S350			S2648		
	*n*^d^	*R*_*P*_	RMSE	*n*^d^	*R*_*P*_	RMSE
TNet-MP-2	350	0.81	0.94	2648	0.77	0.94
STRUM^b^	350	0.79	0.98	2647	0.77	0.94
TNet-MP-1	350	0.74	1.07	2648	0.72	1.02
mCSM^b^^,^^c^	350	0.73	1.08	2643	0.69	1.07
INPS^b^^,^^c^	350	0.68	1.25	2648	0.56	1.26
PoPMuSiC 2.0^b^	350	0.67	1.16	2647	0.61	1.17
PoPMuSiC 1.0^a^	350	0.62	1.23	-	-	-
I-Mutant 3.0^b^	338	0.53	1.35	2636	0.60	1.19
Dmutant^a^	350	0.48	1.38	-	-	-
Automute^a^	315	0.46	1.42	-	-	-
CUPSAT^a^	346	0.37	1.46	-	-	-
Eris^a^	334	0.35	1.49	-	-	-
I-Mutant 2.0^a^	346	0.29	1.50	-	-	-

Comparison of Pearson correlation coefficients (*R*_*P*_) and RMSEs (kcal/mol) of various methods on the prediction task of the “S350” set and 5-fold cross validation of the “S2648”. TNet-MP-1 is our multichannel topological convolutional neural network model that solely utilizes topological information. TNet-MP-2 is our model that complements TNet-MP-1 with auxiliary features.

*^a^* Data directly obtained from Worth *et al* [89].

*^b^* Data obtained from Quan *et al* [94].

*^c^* The results reported in the publications are listed in the table. According to Ref. [94], the data from the online server has *R*_*p*_ (RMSE) of 0.59 (1.28) and 0.70 (1.13) for INPS and mCSM respectively in the task of S350 set.

*^d^* Number of samples successfully processed.

### Multi-task deep learning prediction of membrane protein mutation impacts

Multi-task learning offers an efficient way to improve the predictions associated with small data sets by taking the advantage of other larger data sets [97]. Although a large amount of thermodynamic data is available for globular protein mutations, the mutation data set for membrane proteins is relatively small, between 200 and 300 proteins [98]. The small size of membrane protein mutation data limits the success of data driven approaches, such as ensemble of trees. While the popular multi-task learning framework built on linear regression with regularization techniques lacks the ability to extract the relationship between very low level descriptors and the target quantity. A neural network with a hierarchical structure provides a promising option for such problems. We add the prediction of globular protein stability changes upon mutation as an auxiliary task for the prediction of membrane protein stability changes upon mutation. In the designed network architecture, two tasks share convolution layers and the network splits into two branches with fully connected layers for the two tasks. Intuitively, the task of globular protein mutation predictions help to extract higher level features from low level topological representations. Thus, the branch for membrane protein mutation predictions learns the feature-target relationship from the learned high level features.

The proposed method is tested on a set of 223 mutation instances of membrane proteins covering 7 protein families named “M223” data set [98] with 5-fold cross validation. A comparison with other methods is shown in Table 3. TNet-MMP-1 employes multichannel topological convolutional neural networks with topological features from the “M223” data set, while TNet-MMP-2 is a multi-task multichannel topological convolutional neural network (MM-TCNN) architecture. Unlike TNet-MP-2, both TNet-MMP-1 and TNet-MMP-2 do not use auxiliary features. Our goal is to test the performance of the multi-task architecture on the improvement of high level feature extraction from low level features. Pearson correlation coefficient of membrane protein mutation prediction is improved by 9.6%, i.e., from 0.52 to 0.57 by the multi-task algorithm that trains and predicts the present “M223” data set with the “S2648” date set. As noted by Kroncke *et al*, there is no reliable methods for the prediction of membrane protein mutation impacts at the present [98]. Our TNet results, though not satisfactory, are the best among the methods tested on this problem.

**Table 3:** Performance comparisons of TNet-MMP and other methods.

Method	*R*_*P*_	RMSE
TNet-MMP-2^d^	0.57	1.09
TNet-MMP-1^c^	0.52	1.15
Rosetta-MP	0.31	-
Rosetta (High)^a^	0.28	-
FoldX	0.26	2.56
PROVEAN	0.26	4.23
Rosetta-MPddG	0.19	-
Rosetta (low)^b^	0.18	-
SDM	0.09	2.40

Comparison of Pearson correlation coefficients (*R*_*P*_) and RMSEs (kcal/mol) on 5-fold cross validation for the “M223” data set for various methods. Except for the present results for TNet-MMP-1 and TNet-MMP-2, all other results are adopted from Kroncke *et al* [98]. The results of Rosetta methods are obtained from Fig. S1 of Ref. [98] where RMSE is not given. The results of other methods are obtained from Table S1 of Ref. [98]. Many less competitive results of the machine learning based methods reported in Ref. [98] are not listed since these servers were not machine learning based. Among the methods listed, only Rosetta methods have terms describing the membrane protein system and other methods are not specifically tuned for membrane proteins.

*^a^* High resolution.

*^b^* Low resolution.

*^c^* The multichannel topological convolutional neural network architecture with topological features from “S223” data set.

*^d^* The multi-task multichannel topological convolutional neural network (MM-TCNN) architecture trained with an auxiliary task of globular protein prediction using the “S2648” data set.

## Discussion

The adoption of convolutional neural network concepts in this work is motivated by the underlying spatial relationship along the distance scale (filtration) dimension. Properties that reside in different distance scales are heterogeneous so unlike images or videos, there is no obvious transferable property of the convolution filters along the convolution dimension in the proposed method. To take this into consideration, the convolution layers are substituted with “locally connected layers”, where the local connection properties are conserved whilst the filters applied to different distance scales are allowed to be different. The RMSE is in kcal/mol for the mutation problems and pKd/pKi units for the protein-ligand binding problem. The performance in *R*_*P*_ (RMSE) significantly decreases from 0.81 (0.94) to 0.77 (1.02) for the task of “S350” set prediction in the mutation impact example. This shows that the construction of lower level features in the lower sparse layers benefits from sharing filters along the distance scale and indicates the existence of some common rules for feature extractions at different distance scales.

Intuitively, the dimension 0 inputs describe pairwise atomic interactions, which clearly contribute to the prediction of the target properties. In contrast, dimension 1 and dimension 2 topological features characterize the hydrophobic network and geometric rings and voids. To understand to what extent the higher topological dimensions help the characterization of biomolecules, we separate the dimension 0 inputs from higher dimensional inputs in the prediction of “S350” set in the mutation impact on protein stability example and in the protein-ligand binding affinity prediction for v2007 set example. To compare the performance of different sets of features, 50 single models are trained for each feature set. Twenty of the 50 trained models are randomly chosen and bagged, and this procedure is repeated 100 times with the median results reported. The individual performances measured by *R*_*P*_ (RMSE) for dimension 0 features are 0.73 (1.09) and 0.82 (1.40), respectively for the mutation and binding predictions. For dimensions 1 and 2 features, *R*_*P*_ (RMSE) are 0.66 (1.21) and 0.78 (1.54), respectively for the mutation and binding predictions. The combination of all dimension features results in better *R*_*P*_ (RMSE) of 0.74 (1.08) and 0.83 (1.37), respectively for the mutation and binding predictions, showing that two sets of features both contribute to predictions. The alpha complex is used for geometric characterization and therefore is in $R^{3}$ with Betti number up to dimension 2. It is possible that the higher dimensional Betti numbers in a more abstract setup such as Vietoris-Rips complex for the characterization of an interaction network will enrich the representation and deliver improved results.

**Fig 1:**
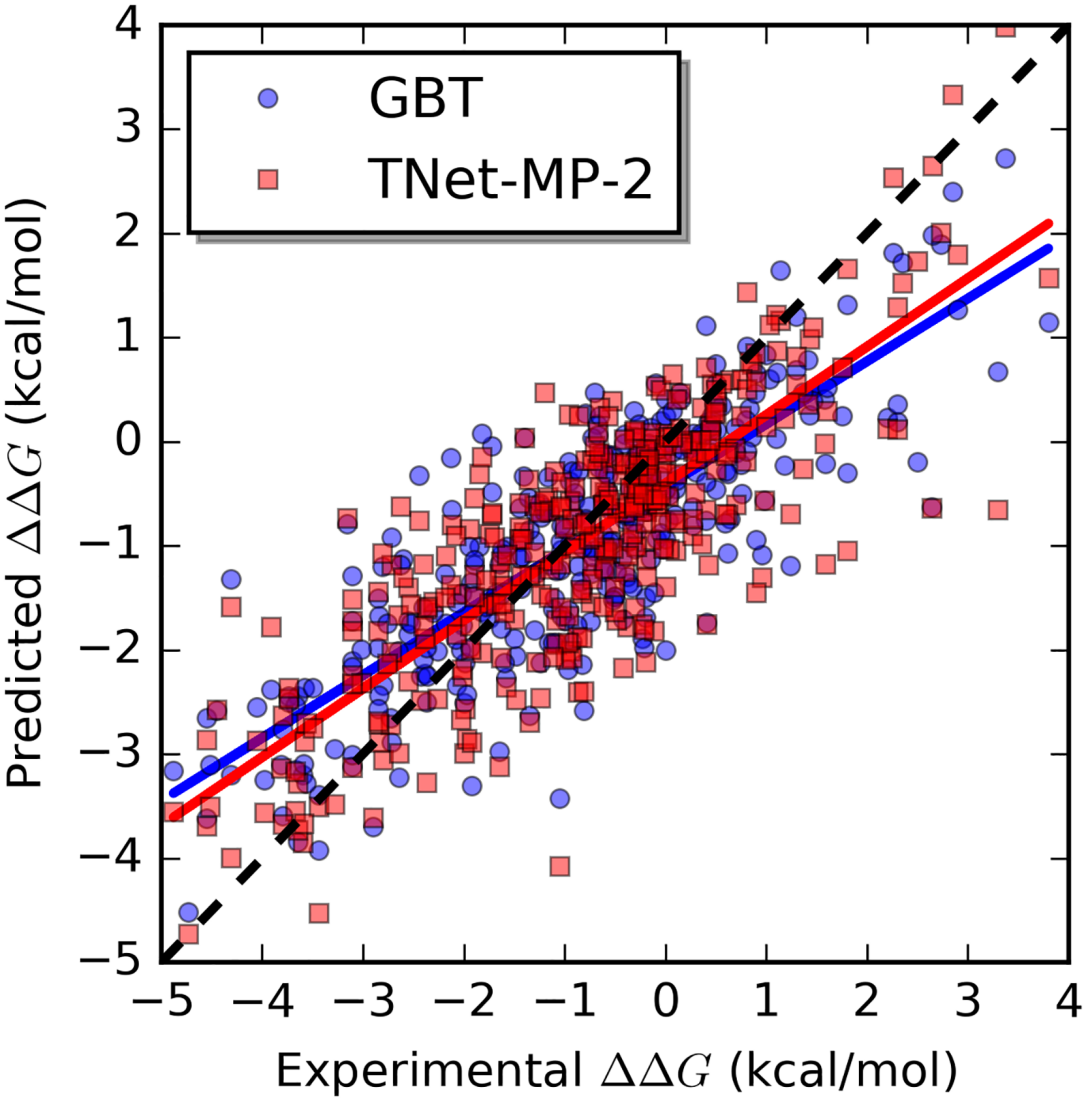
A comparison of behaviors of the GBT based method and the neural network based method. The plot is for the prediction task of the S350 dataset. The linear fit for GBT prediction [60] is *y* = 0.603*x* − 0.435 and for TNet-MP-2, *y* = 0.657*x* − 0.422.

Another popular class of machine learning methods is the ensemble of trees methods. Many modern methods for biomolecular property prediction are based on random forest (RF) and gradient boosting trees (GBTs). The ensemble of decision trees has the capability of learning complicated functions, but GBTs learn to partition the feature space based on the training data which means that they do not have the ability to appropriately extrapolate the learned function to broader situations than the provided training data. Additionally, it is generally the case that data samples are unevenly distributed. It has been observed that in many applications, where among the dataset, there are just a handful of samples with large absolute value for the target property, methods of ensembles of trees tend to overestimate (underestimate) the border cases with very negative (positive) target values. The neural network, due to its different ways of learning the underlying function, seems to be able to deliver better results for the border cases. Therefore, similar to the idea of bagging, methods of ensembles of trees and neural network based methods may result in different error characteristics for different samples and can potentially improve the predictive power by correcting each others’ error when the results from different models are averaged. In the example of prediction of the “S350” set, we obtained performance of 0.82 (0.92) for *R*_*P*_ (RMSE) in our other work using handcrafted features with gradient boosting trees [60]. When the results are averaged for the two methods, the performance is improved to 0.83 (0.89) which is better than both individual methods. Similar improvement is observed for the protein-ligand binding example with v2007 set. Our method based on handcrafted features and gradient boosting trees with performance 0.82 (1.40) [61] and the method presented in this work with performance 0.83 (1.37) can achieve improved performance of 0.84 (1.35) when the two results are combined by averaging. An intuitive illustration is shown in Fig 1. It can be seen from the plot that the neural network based method presented in this work performs better than the GBT based method for samples with high ΔΔ*G* or with low ΔΔ*G*. The slope of linear fitting of the predicted values to the experimental data is 0.66 for the neural network based method and 0.60 for the GBT based method which also illustrates that the neural network based method handles border cases better. The observed improvement is marginal since it is mainly on a small portion of the samples.

In conclusion, the approach introduced in this work utilizes element-specific persistent homology to efficiently characterize 3D biomolecular structures in terms of multichannel topological invariants. Convolutional neural network facilitates the automatic feature extraction from multichannel topological invariant inputs. The flexible and hierarchical structure of neural network allows seamless combination of automatically extracted features and handcrafted features. It also makes it easy to implement multi-task learning by combining related tasks to a desired level of model sharing by tuning the layer of model branching. The proposed topology based neural network (TopologyNet) methods have been shown to outperform other existing methods in protein-ligand binding affinity predictions and mutation induced protein stability change predictions. The proposed methods can be easily extended to other applications in the structural prediction of biomolecular properties. They have the potential to further benefit from the fast accumulating biomolecular data. The combination of the proposed methods and existing RF and GBT based methods is expected to deliver improved results.

## Methods

In this section, we give a brief explanation of persistent homology before introducing topological representations of protein-ligand binding and protein changes upon mutation. Multichannel topological deep learning and multi-task topological deep learning architectures are constructed for binding affinity and mutation impact predictions. The source codes with examples of feature construction for the binding problem and the mutation problem are in S1 Code. Binding topological features and S2 Code. Mutation topological features respectively. The network architectures, parameters, and training procedures are listed in S2 Text. Network architectures. The description of the auxiliary features together with pseudocode for the mutation application are listed in S1 Text. Handcrafted features.

### Persistent homology

Simplicial homology gives a computable way to distinguish one space from another in topology and is built on simplicial complexes which can be used to extract topological invariants in a given data set. A simplicial complex *K* is a topological space that is constructed from geometric components of a data set, including discrete vertices (nodes or atoms in a protein), edges (line segments or bonds in a biomolecule), triangles, tetrahedrons and their high dimensional counterparts, under certain rules. Specifically, a 0-simplex is a vertex, a 1-simplex an edge, a 2-simplex a triangle, and a 3-simplex represents a tetrahedron. The identification of connectivity of a given data set can follow different rules which leads to, for example, Vietoris-Rips (VR) complex, Čech complex and alpha complex. The linear combination of *k*-simplexes is called *k*-chain, which is introduced to associate the topological space, i.e., simplicial complex, with algebra groups, which further facilitate the computation of the topological invariants (i.e., Betti numbers) in a given data set. Specifically, the set of all *k*-chains of a simplicial complex *K* are elements of a chain group, which is an abelian group with a modulo-2 addition operation rule. Loosely speaking, a boundary operator systematically eliminates one vertex from the *k*-simplex at a time, which leads to a family of abelian groups, including the *k*th cycle group and the *k*th boundary group. The quotient group of the *k*th cycle group and the *k*th boundary group is called the *k*th homology group. The *k*th Betti number is computed for the rank of the *k*th homology group.

Persistent homology is constructed via a filtration process, in which the connectivity of the given data set is systematically reset according to a scale parameter. More specifically, a nested sequence of subcomplexes is defined via a filtration parameter, such as the growing radius of protein atoms located at their initial coordinates. For each subcomplex, homology groups and the corresponding Betti numbers can be computed. Therefore, the evolution of topological invariants over the filtration process can be recorded as a barcode [45] or a persistence diagram. For a given data set, barcodes represent the persistence of its topological features over different spatial scales.

### Topological representation of biomolecules

#### Topological fingerprints

**Fig 2:**
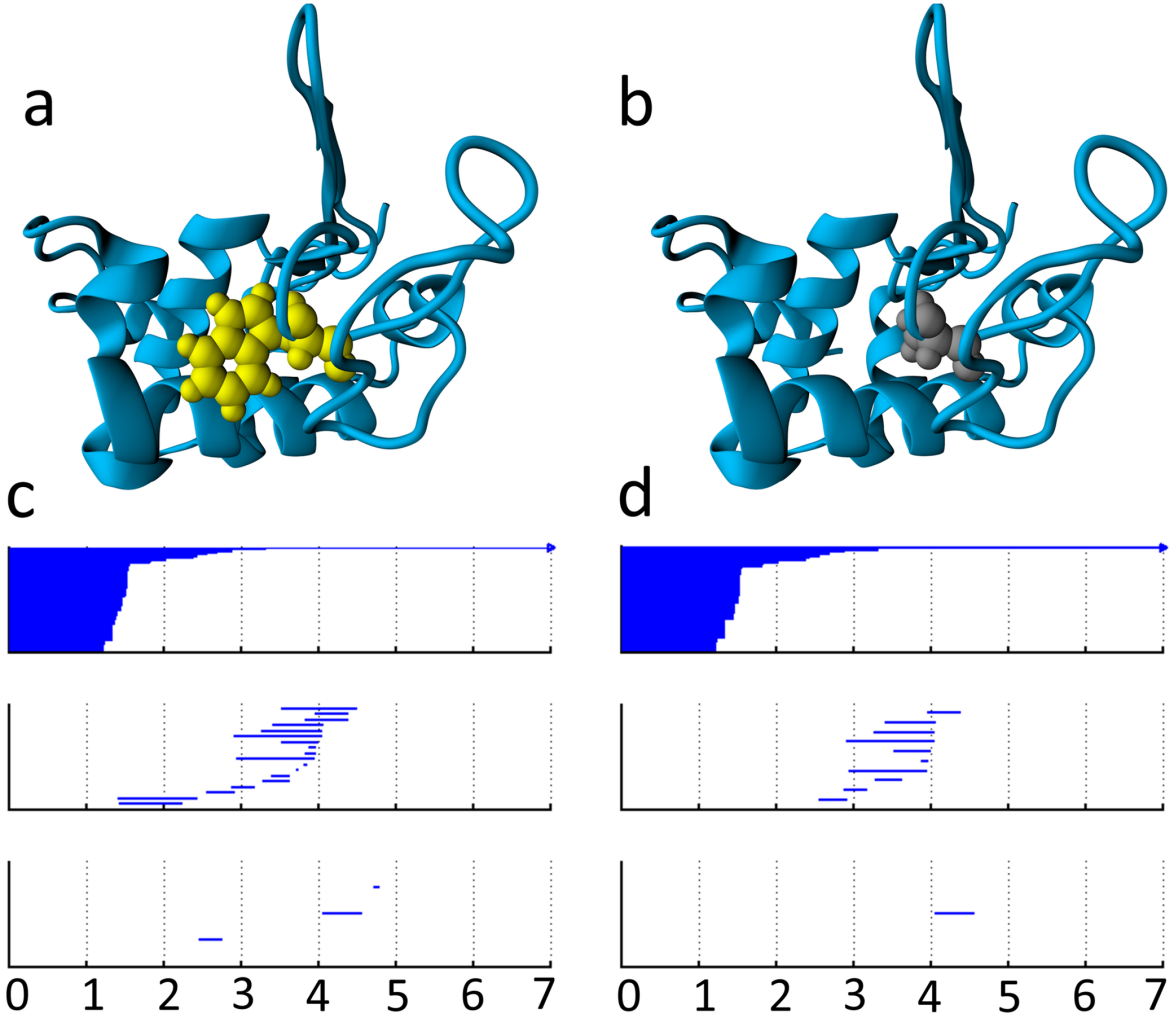
An illustration of barcode changes from wild type to mutant proteins. **a** The wild type protein (PDB:1hmk) with residue 60 as Trp. **b** The mutant with residue 60 as Ala. **c** Wild type protein barcodes for heavy atoms within 6 Å of the mutation site. Three panels from top to bottom are Betti-0, Betti-1, and Betti-2 barcodes, respectively. The horizontal axis is the filtration radius (Å). **d** Mutant protein barcodes obtained similarly to those of the wild type.

A basic assumption of persistent homology as applied to biomolecular function prediction is that 1D biomolecular persistent barcodes are able to effectively characterize 3D biomolecular structures. We call such barcodes topological fingerprints (TFs) [49, 50]. Fig 2 illustrates the TFs of a wild type protein (PDB:1hmk) and its mutant obtained from persistent homology calculations using the VR complex. The mutation (W60A) occurred at residue 60 from Trp to Ala is shown at Fig 2a and 2b. A large residue (Trp) at the protein surface is replaced by a relatively small one (Ala). The corresponding barcodes are given in Fig 2c and 2d, where three panels from top to bottom are for Betti-0, Betti-1, and Betti-2, respectively. The barcodes for the wild type are generated using heavy atoms within 6Å from the mutation site. The mutant barcodes are obtained with the same set of heavy atoms in the protein except for those in the mutated residue. In two Betti-0 panels, the difference in the number of bars is equal to the difference in the number of heavy atoms between the wild type and mutant. Broadly speaking, the lengths of short bars reflect the bond length of the corresponding heavy atom. Therefore, in both the wild type protein and the mutant, bond lengths for most heavy atoms are smaller than 1.8Å. Additionally, bars that end between 1.8Å and 3.8 Å might correlate with hydrogen bonds. Comparing **c** and **d**, one can easily note the increase in the number of bars that end in the range of 1.8–3.8 Å in the mutant, which indicates a less compact atom arrangement. In Betti-1 and Betti-2 panels, the mutant has fewer bars than the wild type does because a smaller surface residue at 60 creates fewer ring and cavity contacts with the rest of the protein.

#### Element-specific persistent homology

The all heavy atom topological representation of proteins does not provide enough biological information about protein structures, such as bond length distribution of a given type of atoms, hydrogen bonds, hydrophobic and hydrophilic effects, etc. Therefore, we use the element-specific topological fingerprint (ESTF) to offer a more detailed characterization of protein-ligand binding and protein mutation. For example, Betti-1 and Betti-2 ESTFs from carbon atoms are associated with hydrophobic interaction networks in biomolecules. Similarly ESTFs between nitrogen and oxygen atoms correlate to hydrophilic interactions and/or hydrogen bonds in biomolcules. However, hydrogen atoms are typically absent from structures in the PDB and thus are not used in our data driven ESTF description. For proteins, commonly occurring heavy atom types include C, N, O, and S. For ligands, we use 9 commonly occurring atom types, namely C, N, O, S, P, F, Cl, Br, and I. To characterize the interactions between protein and ligand binding, we construct cross protein-ligand ESTFs such that one type of heavy atoms is chosen from the protein and the other from the ligand. Therefore, there are a total of 36 sets of ESTFs in each topological dimension. For mutation characterization, we describe the interactions between mutated residue and the rest of the protein and arrive at 9 sets of ESTFs in each topological dimension considering { C, N, O } for protein atoms. Similarly, we generate 9 sets of cross ESTFs in each topological dimension from the wild type protein to study the interactions between the residue to be mutated and the rest of the protein. However, high dimensional Betti-1 and Betti-2 invariants require the formation of high order complexes. As non-carbon atoms do not occur very often, Betti-1 and Betti-2 ESTFs are generated for all carbon atoms or all heavy atoms, except specified.

The TFs and ESTFs are originally stored as collections of barcodes denoted by B(α,C,D) with *α* labeling the selection of atoms depending on atom types and affiliations (i.e., protein, ligand or mutated residue). C denotes the type of simplicial complex (i.e., VR complex or alpha complex) and D indicates the dimension, such as Betti-0, Betti-1, or Betti-2. A collection of barcodes can have any number of barcodes and thus can not be directly fed to deep learning models. Additionally, as shown in Fig 2, it is important to keep track of the birth, death, and persistence patterns of the barcodes, because this information is associated with the bond length, ring or cavity size, flexibility and steric effect. Moreover, Jeffrey suggested that there are strong, moderate and weak hydrogen bond interactions with donor-acceptor distances of 2.2-2.5Å, 2.5-3.2Å, and 3.2-4.0Å, respectively [99]. To this end, we construct structured vectors **V**^b^, **V**^d^, and **V**^p^ to respectively describe the birth, death, and persistent patterns of the barcodes in various spatial dimensions. Practically, the filtration interval [0, *L*] is divided into *n* equal length subintervals and the patterns are characterized on each subinterval. The description vectors are defined as

$$\begin{array}{c} \begin{array}{cc} V_{i}^{b} & =∥\{\left(b_{j},d_{j}\right)\in B\left(\alpha ,C,D\right)|\left(i-1\right)L/n\leq b_{j}\leq iL/n\}∥,\;1\leq i<n,\\ V_{i}^{d} & =∥\{\left(b_{j},d_{j}\right)\in B\left(\alpha ,C,D\right)|\left(i-1\right)L/n\leq d_{j}\leq iL/n\}∥,\;1\leq i<n,\\ V_{i}^{p} & =∥\{\left(b_{j},d_{j}\right)\in B\left(\alpha ,C,D\right)|\left(i-1\right)L/n\geq b_{j},\;iL/n\leq d_{j}\}∥,\;1\leq i\leq n, \end{array} \end{array}$$ (1)

where ‖⋅‖ is cardinality of sets. Here *b*_*j*_, *d*_*j*_ are birth and death of bar *j*. The three types of representation vectors are computed for sets of Betti-1 and Betti-2 bars. For Betti-0 bars, since their birth positions are uniformly 0, only **V**^d^ needs to be addressed. To characterize pairwise interactions between atoms, it is convenient to simply use pairwise distance information between atoms. The corresponding image-like representation, denoted by **V**^r^, can be constructed similarly to **V**^d^ by substituting the set of barcodes by a collection of distances between the atom pairs of interest. It should be noted that **V**^r^ is not equivalent to **V**^d^ in most simplicial complex setups. Generally speaking, **V**^r^ also reflects the 0th order topological connectivity information. It is used as the characterization of 0th order connectivity of the biomolecules in the applications shown in this work. Finally, we let *X*_*s*_ denote all the feature vectors for the *s*th sample and let *Y*_*s*_ denote the corresponding target value.

#### Image-like multichannel topological representation

To feed the outputs of TFs into the convolutional neural network, the barcodes are transformed to a 1D-image-like representation with multiple channels. Topological feature vectors, **V**^b^, **V**^d^, and **V**^p^, can be viewed as one-dimensional (1D) images. Each subinterval in the filtration axis represents a digit (or pixel) in the 1D-image-like representation. Such a treatment of topological features describes the topological information with appropriately chosen resolution of *L*/*n*. Meanwhile, the chemical information in the ESTFs of B(α,C,D) are described by multiple channels in the 1D-image-like representation, which is similar to the RGB color image representation. However, in our description, each pixel is associated with *m* channels to describe different element type, protein mutation status (i.e., wild type and mutant), topological dimension (i.e., Betti-0, Betti-1 and Betti-2), and topological event (i.e., birth, death, and persistence). Each element in the 1D-image-like representation is standardized to have zero mean and unit variance among the data sets. This 1D-image-like topological representation can be easily transferred among problems such as protein-ligand binding affinity modeling and prediction of protein stability change upon mutation. Traditional machine learning approaches require manual extraction of features for each domain of application. When the convolutional neural network is applied, the convolution layers identify local patterns of atomic interactions and the fully connected layers then extract higher level descriptions of the system by combining local patterns at various distance scales.

#### Multichannel topological invariants for protein-ligand binding prediction

**Fig 3:**
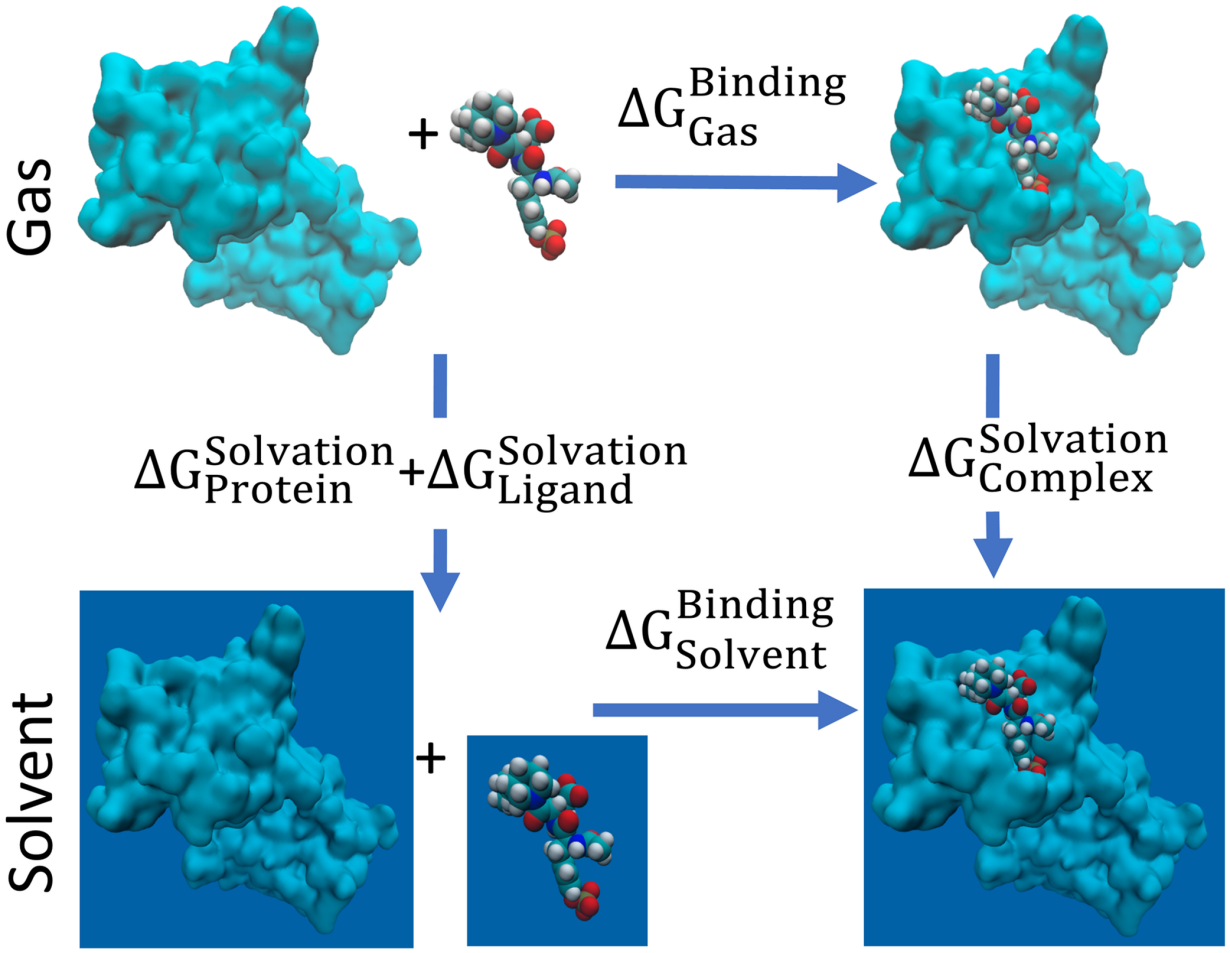
Energy cycle of protein-ligand binding free energy modeling.

In computation, the binding affinity, or alternatively the binding free energy, can be modeled via an energy cycle as shown in Fig 3 where the main contributors to the process are intermolecular interactions and solvation effects. In this work, we consider the set of element types $L^{e}=\left\{C,N,O,S,P,F,\text{Cl},\text{Br},I\right\}$ contained in ligands and $P^{e}=\left\{C,N,O,S\right\}$ contained in proteins. We define an opposition distance between two atoms *a*_*i*_ and *a*_*j*_ as

$$\begin{array}{c} d^{op}\left(a_{i},a_{j}\right)=\{\begin{array}{cc} d(a_{i},a_{j}) & ,A\left(a_{i}\right)\neq A\left(a_{j}\right)\\ \infty & ,A\left(a_{i}\right)=A\left(a_{j}\right) \end{array} \end{array},$$ (2)

where *d*(⋅, ⋅) is Euclidean distance between two atoms and *A*(⋅) denotes the affiliation of an atom which is either a protein or a ligand.

The ESTFs used in this application are summarized in Table 4. The structured description vectors of the ESTFs are generated according to the definition given in Eq (1). As shown in Table 4, five sets of ESTFs are constructed. The differences between the description vectors arising from Set 2 and Set 3, and between those arising from Set 4 and Set 5 are also employed as representation vectors to address the impact of ligand binding resulting in a total of 72 representation vectors (i.e., channels) forming the 1D-image-like representation of the protein-ligand complex. Pairwise interactions are characterized for the 36 element pairs with {C, N, O, S} for the protein and {C, N, O, S, F, P, Cl, Br, I} for the ligand with **V**^*d*^ providing 36 channels. The birth (**V**^*b*^), death (**V**^*d*^), and persistence (**V**^*p*^) for Betti-1 and Betti-2 barcodes are computed for carbon atoms and all heavy atoms of the protein and the protein-ligand complex which results in 24 channels. The difference between the characterization of the protein and the protein-ligand complex accounts for another 12 channels. Thus, we have a total of 72 channels. Here, 0-dimensional TFs describe intramolecular interactions between the protein and ligand. All heavy atom TFs delineate the geometric effect of protein-ligand binding. The TFs of carbon atoms account for hydrophobic effects and also implicitly reflect the solvation effects. The distance scale interval, [0, 50] Å is divided into bins of length 0.25 Å.

**Table 4:** Topological representations of protein-ligand complexes.

Set	Atoms used	Distance	Complex	Dimension
1	$\left\{a\in P|T\left(a\right)=e_{P}\right\}\cup \left\{a\in L|T\left(a\right)=e_{L}\right\},e_{P}\in P^{e},e_{L}\in L^{e}$	*d*^*op*^	-	0
2	$\left\{a\in P|T\left(a\right)\in P^{e}\right\}$	Euclidean	Alpha	1,2
3	$\left\{a\in P|T\left(a\right)\in P^{e}\right\}\cup \left\{a\in L|T\left(a\right)\in L^{e}\right\}$	Euclidean	Alpha	1,2
4	{a∈P|T(a)=C}	Euclidean	Alpha	1,2
5	{a∈P|T(a)=C}∪{a∈L|T(a)=C}	Euclidean	Alpha	1,2

P and L are sets of atoms in protein and in ligand. *T*(⋅) denotes element type of an atom. *e*_P_ is an element type in protein and *e*_L_ is an element type in ligand. “Complex” refers to the type of simplicial complex used and “Dimension” refers to the dimensionality of a topological invariant.

#### Multichannel topological invariants for the prediction of protein folding free energy change upon mutation

**Fig 4:**
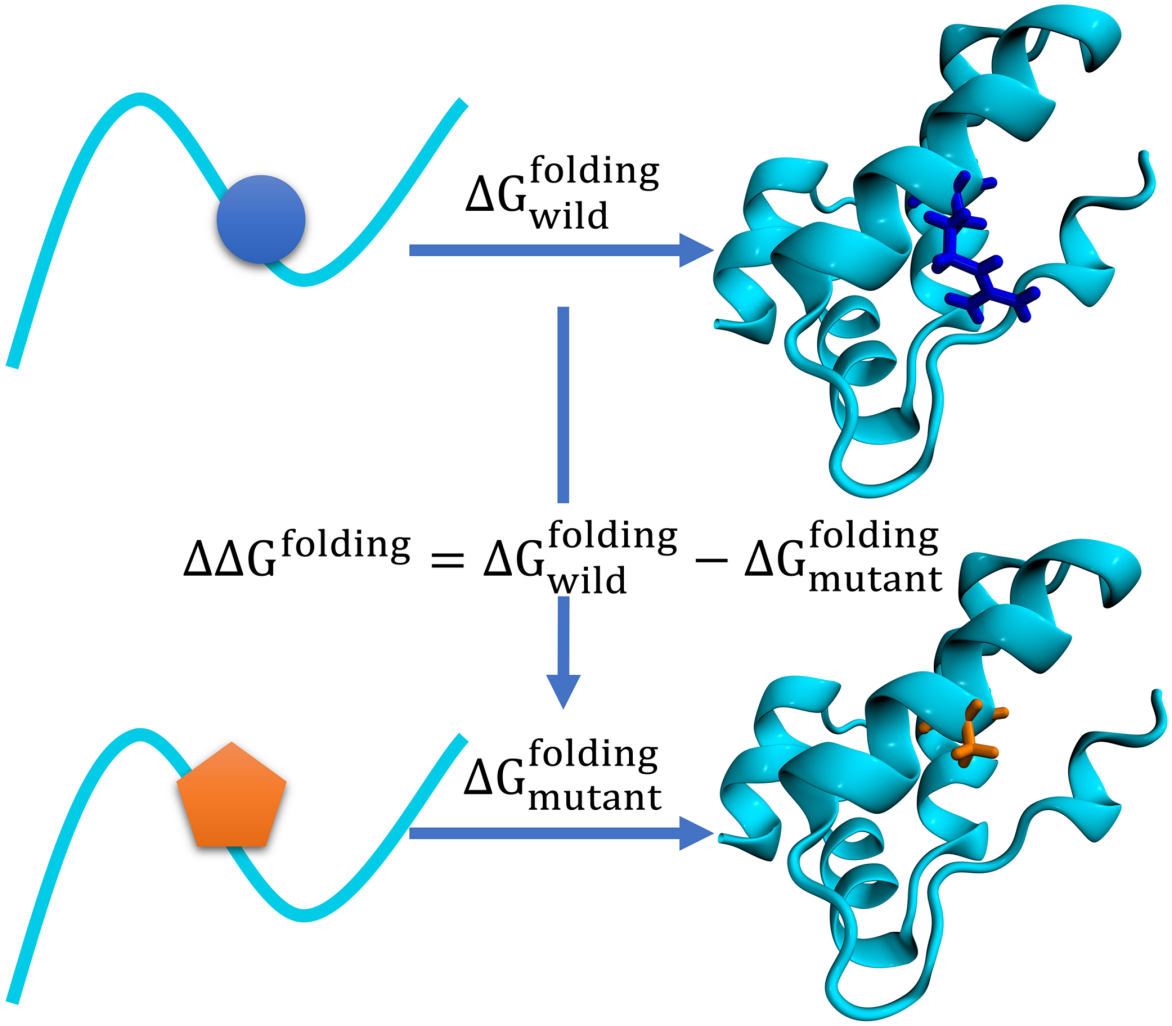
Mutation induced protein folding free energy changes.

Modeling protein folding free energy change upon mutation basically involves the unfolded states and folded structures of the mutant and the wild type as shown in Fig 4. Since unfolded states of proteins are highly dynamic which significantly increases the modeling cost due to the need of sampling over large conformation space, we only analyze the folded states of the mutants and the wild type proteins in this application. Similar to the protein-ligand binding affinity prediction, atomic interactions between specific element types, geometric effects, and hydrophobic effects are characterized. The persistent homology analysis performed in this application is summarized in Table 5. The differences between the description vectors arising from Sets 1 and 2, and between those arising from Sets 3 and 4 are also included to account for changes caused by mutation. The 1D-image-like representation in this application thus has a channel size of 45. The pairwise interaction pattern is characterized for 9 element pairs from the element set {C, N, O }. For example, the interactions between the carbon atoms of the mutation site and the nitrogen atoms from the rest of the protein. Such characterization for mutant protein, wild protein, and the difference between these characterizations account for 27 channels. The birth, death, and bar persistence are characterized for Betti-1 and Betti-2 barcodes for all heavy atoms of both the wild type protein and the mutant protein resulting in 12 channels. The difference between the mutant and the wild type, which accounts for 6 channels, is also included. Thus, we have a total of 45 channels. The distance scale interval, [0, 12] Å is divided into bins of length 0.25 Å. An example of the persistent homology barcodes of a mutant and its wild type is given in Fig 2.

**Table 5:** Topological representations for protein mutation problem.

Set	Atoms selected	Distance	Complex	Dimension
1	$\{a\in P^{W}\backslash M^{W}\left|T\left(a\right)=e_{P}\right\}\cup \left\{a\in M^{W}|T\left(a\right)=e_{M}\right\},e_{P},e_{M}\in P^{e}$	*d*^*op*^	-	0
2	$\{a\in P^{M}\backslash M^{M}\left|T\left(a\right)=e_{P}\right\}\cup \left\{a\in M^{M}|T\left(a\right)=e_{M}\right\},e_{P},e_{M}\in P^{e}$	*d*^*op*^	-	0
3	$\left\{a\in P^{W}|T\left(a\right)\in P^{e}\right\}$	Euclidean	Alpha	1,2
4	$\left\{a\in P^{M}|T\left(a\right)\in P^{e}\right\}$	Euclidean	Alpha	1,2

Here $P^{W}$, $P^{M}$, $M^{W}$, and $M^{M}$ are sets of atoms of wild type protein, mutant protein, mutation site in the wild type protein, and mutated site in the mutant protein. Here $P^{e}=\left\{C,N,O\right\}$ and *T*(⋅) is the same as defined in Table 4. The distance function *d*^*op*^ is similar to the one defined in Eq (2), while the affiliation function *A*(⋅) returns either M or P\M.

### Multichannel topological convolutional neural network

**Fig 5:**
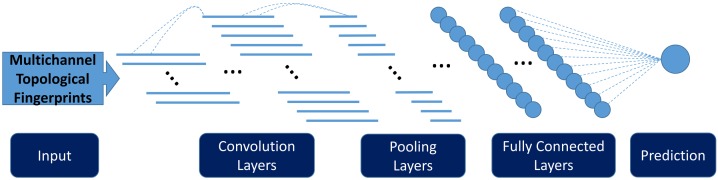
An illustration of the 1D convolutional neural network. The network consists of repeated convolution layers and pooling layers followed by several fully connected layers.

The preprocessed multichannel topological image is standardized with mean 0 and standard deviation 1 for use in the convolutional neural network. A convolutional neural network with a few 1D convolution layers, followed by several fully connected layers, is used to extract higher level features from multichannel topological images and to perform regression with the learned features. An illustration of the convolutional neural network structure is shown in Fig 5. A brief review of multichannel topological convolutional neural network concepts is given in the case of 1D-image-like inputs. Convolution operation, optimization method for feedforward neural networks, and dropout out technique which prevents overfitting are discussed. One of the advantages of multichannel topological convolutional deep neural networks is their ability to extract features hierarchically from low level topological representations.

#### Convolution operation

Consider an *n* × *m* second order tensor **V**, where *n* is the number of topological feature pixels and *m* is number of channels for each pixel. In this approach, *n* corresponds to the radius filtration dimension of the biomolecular topological analysis and *m* corresponds the number of representation vectors used which are defined in Eq (1). With a predefined window size *w*, a convolutional filter **F** can be represented by a *w* × *m* second order tensor. By moving the window of size *w* along the radius filtration direction of **V**, a sequence of *N*_*f*_ second order tensors, which are subtensors of *V*, are obtained and can be concatenated to form an *N*_*f*_ × *w* × *m* third order tensor **T**. The filter **F** operated on **T** results in a first order tensor **T**_*ijk*_**F**_*jk*_ by tensor contraction. Concatenating the outputs of *n*_*f*_ filters gives an *N*_*f*_ × *n*_*f*_ second order tensor. Generally speaking, a 1D convolution layer takes an *n* × *m* tensor and outputs an *N*_*f*_ × *n*_*f*_ tensor.

#### Optimization

Feedforward neural networks are usually trained by backpropagation where the error of the output layer is calculated and is propagated backward through the network to update its weights. For structured neural networks, conventional *L*_2_ minimization does not work. One popular approach of training a neural network is the stochastic gradient decent (SGD) method. Let Θ be the parameters in the network and L(Θ) be the objective function or learning kernel that is to be minimized. SGD method updates Θ_*i*_ to Θ_*i*+1_ from step *i* to step *i* + 1 as

$$\begin{array}{c} \Theta_{i+1}=\Theta_{i}-\tau \nabla_{\Theta }L\left(\Theta_{i};X_{s},Y_{s}\right), \end{array}$$ (3)

where *τ* is the learning rate, *X*_*s*_ and *Y*_*s*_ are the input and target of the *s*th sample of the training set. In practice, the training set (*X*, *Y*) is often split into mini-batches {(*X*_*s*_, *Y*_*s*_)}_*s*∈*S*_. SGD method then goes through each mini-batch instead of going through only one example at a time. When the landscape of the objective function is like a long steep valley, momentum is added to accelerate convergence of the algorithm. The updating scheme can therefore be changed to

$$\begin{array}{c} \begin{array}{cc} \Delta \Theta_{i} & =\Theta_{i}-\Theta_{i-1},\\ \Theta_{i+1} & =\Theta_{i}-\left(1-\eta \right)\tau \nabla_{\Theta }L\left(\Theta_{i};X_{s}^{i},Y_{s}^{i}\right)+\eta \Delta \Theta_{i}, \end{array} \end{array}$$ (4)

where 0 ≤ *η* ≤ 1 is a scalar coefficient for the momentum term.

#### Dropout

Neural networks with several convolution layers and fully connected layers possess a large number of degrees of freedom which can easily lead to overfitting. The dropout technique is an easy way of preventing network overfitting [100]. During the training process, hidden units are randomly chosen to feed zero values to their connected neighbors in the next layer. Suppose that a percentage of neurons at a certain layer are chosen to be dropped during training. Then, in the testing process, the output of this layer is computed by multiplying a coefficient such as 1 − λ, where λ is the dropout rate, to approximate the average of the network after dropout in each training step.

#### Bagging (bootstrap aggregating)

In addition to dropout technique which regularizes each individual model, bagging is a technique to combine the output of several models trained separately by averaging to reduce generalization error. This is based on the assumption that models with randomness in the training process likely make different errors on testing data. Generally, bagging trains different models on different subsets of the training set. Specifically, as neural networks have relatively high underlying randomness caused by factors including the random weights initialization and the random mini-batch partition, it can benefit from bagging even if the individual models are trained on the same dataset. In this work, bagging of neural network models trained individually with the same architecture and training dataset is used.

#### Incorporating non-image-like features

**Fig 6:**
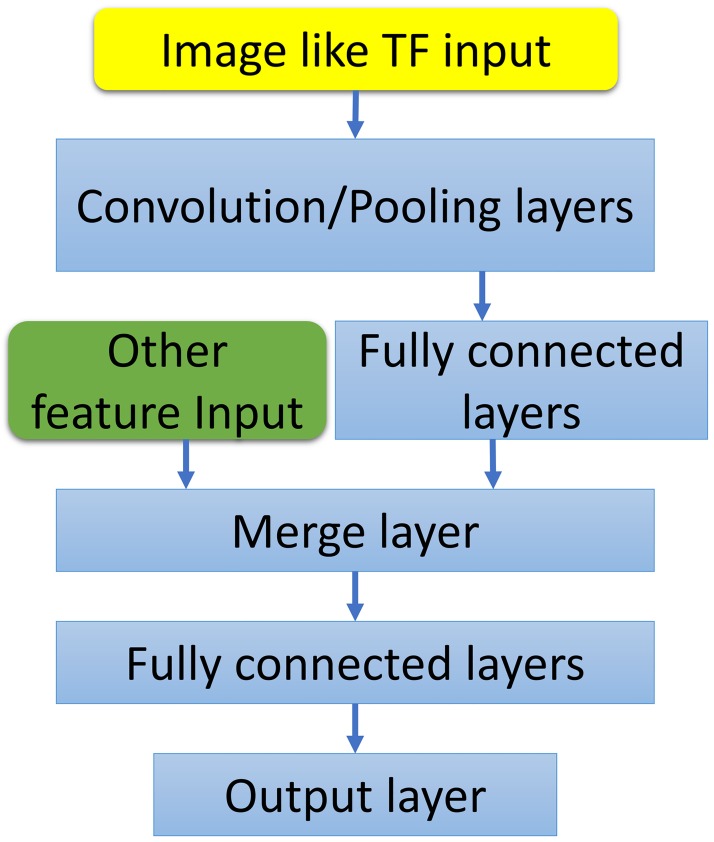
The deep learning architecture for the application to globular proteins. The non-image-like features are incorporated in the multichannel topological convolutional deep neural network by merging the features into the network at one of the fully connected layers.

Deep learning architecture also allows the use of non-image-like features together with image or image-like features. In this work, additional auxiliary features, which are important to mutation analysis, are incorporated after the convolution layers as shown in Fig 6. This approach leads to a 9% improvement to mutation prediction of the “S2648” data set.

#### Multi-task deep learning

**Fig 7:**
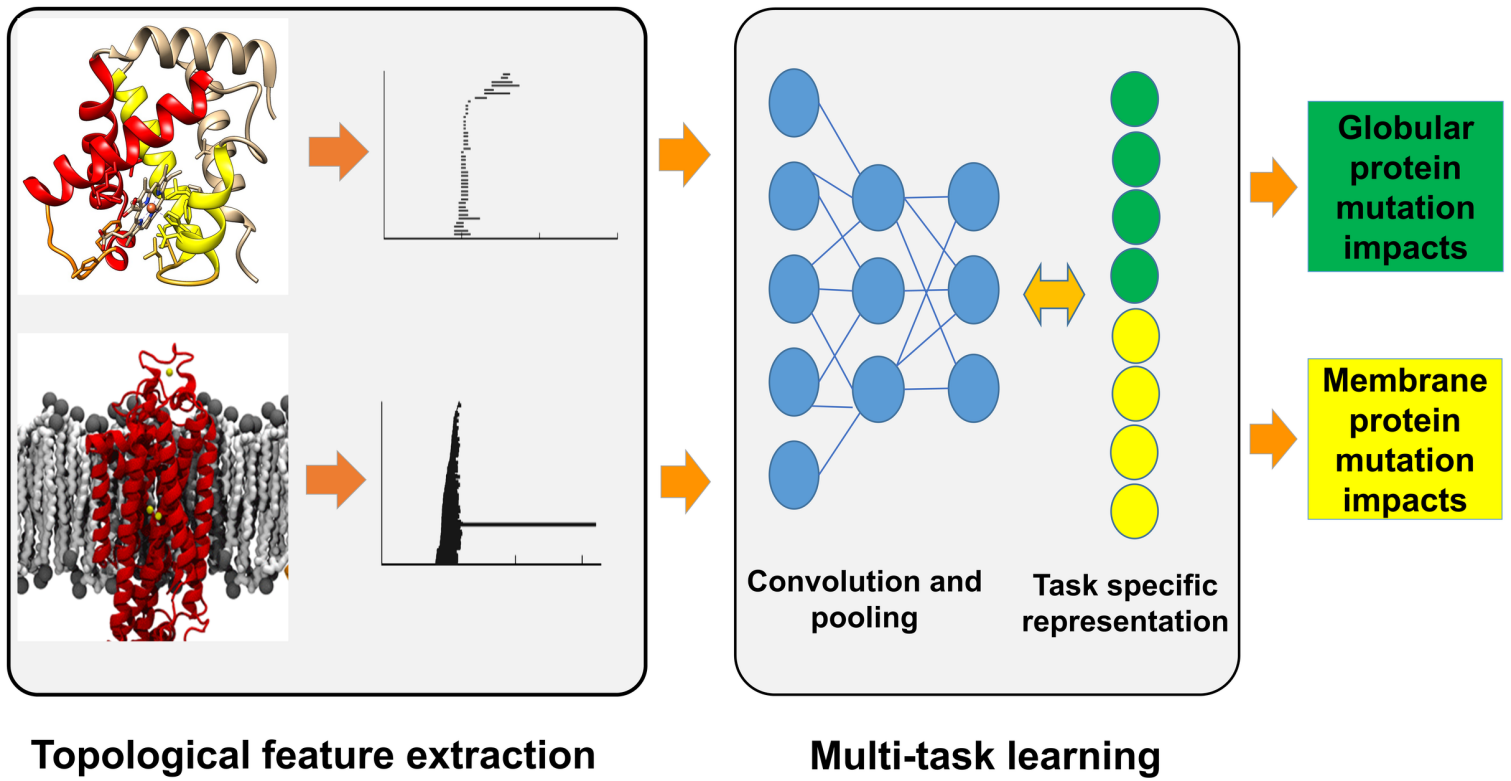
Workflow of the multi-task topological deep learning model. The multi-task multichannel topological convolutional neural network model shares and transforms topological information for the simultaneous training and prediction of globular protein and membrane protein mutation impacts on protein stability.

We construct a multi-task multichannel topological convolutional neural network (MM-TCNN) architecture to carry out simultaneous training and prediction. The common topological attributes and underlying physical interactions in features provide a basis for multi-task predictions. Because the deep neural networks are jointly trained from multiple prediction tasks, we expect the networks to generate robust high-level representations from low level TFs for prediction problems. We also expect that the refined representation would lead to prediction models with improved generalized performance. From the proposed deep learning models, we hope to gain insights into how the nonlinear and nonlocal interactions among topological features impact various prediction tasks, which could further lead to better understanding towards the interactions among biomolecular prediction tasks. Finally, tasks with insufficient training data sets will be more likely to benefit from the information collected from tasks with large training sets in a multi-task learning framework. Fig 7 illustrates our multi-task multichannel topological deep learning architecture for simultaneous training and prediction of globular protein and membrane protein mutation impacts.

**Fig 8:**
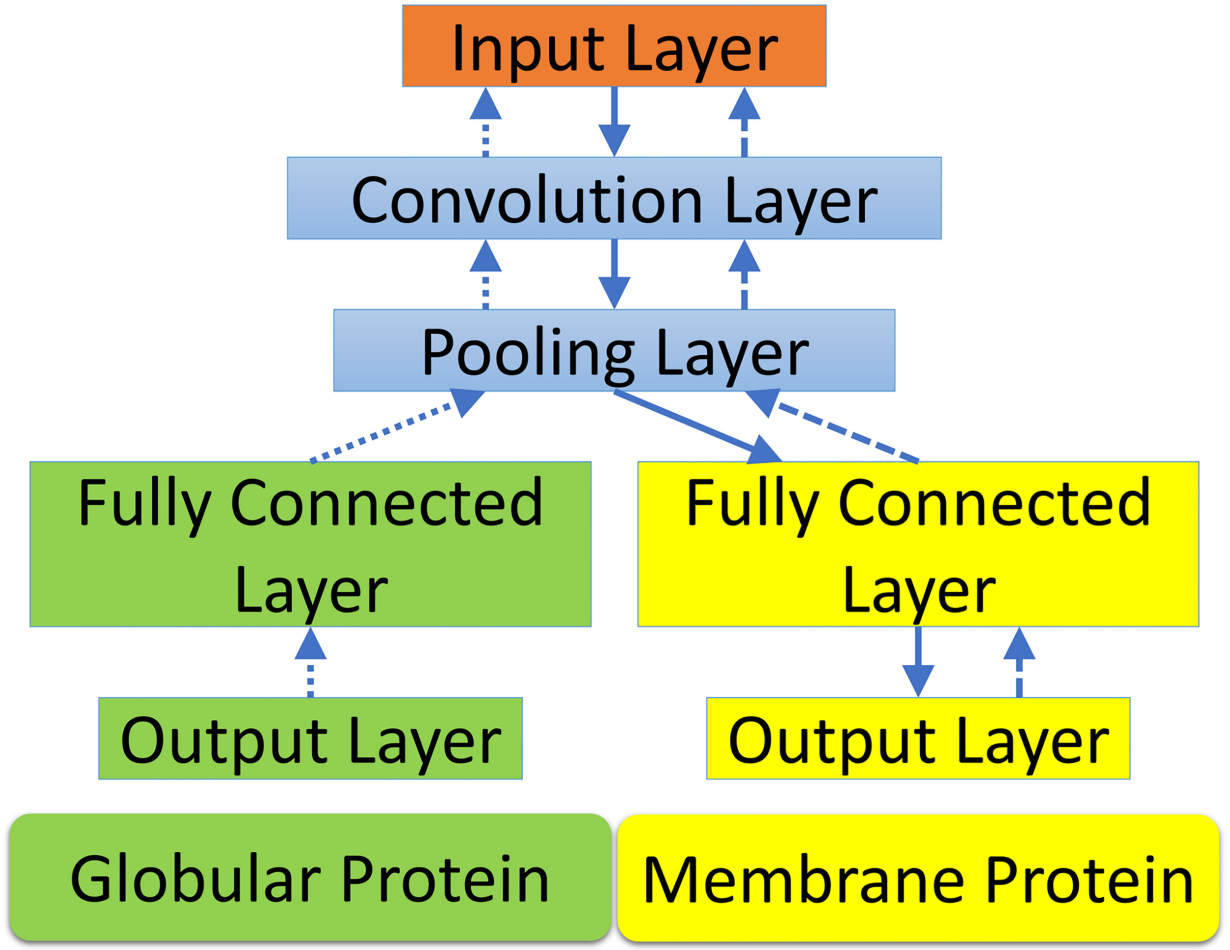
The multi-task deep learning architecture for membrane proteins. Using globular protein stability change upon mutation as an auxiliary task to improve the task of membrane protein mutation prediction. The globular protein stability change upon mutation prediction is used as an auxiliary task to improve the task of predicting membrane protein stability changes upon mutation. The solid arrows show the path of information passing when the model is applied for predictions. The dotted and dashed arrows mark the paths of backpropagation when the network is trained with globular protein data set and membrane protein data set respectively.

In the present mutation analysis, there are two data sets. The mutation data of the large data set for globular proteins are more reliable, while those of the small data set for membrane proteins are noisy and less reliable due to the fact that the current technologies for membrane protein mutagenesis experiments are immature. The prediction for membrane proteins benefits from joint learning with the prediction for globular proteins. The coupling of the two predictions through a neural network is shown in Fig 8.

The general objective function to minimize for multi-task learning through neural networks can be decomposed into training loss, similarity penalty for shared layers, and regularization term as

$$\begin{array}{lll} L\left(\Theta ;X,Y\right) & = & \sum_{j=1}^{N}J_{j}\left(\Theta_{Sj},\Theta_{Bj};X_{j},Y_{j}\right)\\ & + & P\left(\Theta_{S1},\cdots ,\Theta_{SN}\right)\\ & + & R\left(\Theta \right), \end{array}$$ (5)

where Θ is the collection of all parameters to be updated, Θ_*Sj*_ is the set of parameters for the *j*th task of the shared layers, Θ_*Bj*_ is the set of parameters for the *j*th branch of neurons dedicated for the *j*th task, and (*X*_*j*_, *Y*_*j*_) are training data for the *j*th task. Here P is the penalty function which penalizes the difference among *N* sets of parameters. Finally R(·) is the regularization term which prevents overfitting and J is the *j*th loss function. In this work, we force the shared layers of the two problems to be the same and the regularization of the network is realized using dropout.

#### Model training and prediction

Due to the complexity of the network for the mutation example with auxiliary features, a brief parameter search is performed using Hyperopt [101] with only 50 trials allowing flexibility in number of neurons, activation function, and weight initialization. In the protein-ligand binding example, only around 10 sets of parameters are selected manually and tested because of the large input size for the problem.

In the protein-ligand binding affinity predictions, we repeatedly train 100 single neural networks individually. To test the performance of bagging of the models, we randomly select 50 trained models from the 100 individually trained networks and output the average value of the outputs from the 50 selected models as the prediction. The performance is then computed for the bagging. This process is repeated 100 times and both median and best results are reported.

In the mutation induced protein stability predictions, we use the same procedure used in the protein-ligand binding prediction, for the “S350” task, where the training and testing split is predefined. In the case of cross validation, 10 sets of 5-fold splits are generated randomly and 20 single models are generated for each split. The average prediction is taken over the 20 models within each split and the median result of the 10 splits is reported. Bagging of only 20 models is performed here because it is not valid to do bagging of predictors on different cross validation splits. The bagging of 50 models will result in 50(individual models)x10(cross validation splits)x5(five folds) = 2500 training processes which is too computationally expensive. Details of the network architectures of the three examples can be found in Multichannel topological convolutional neural network.

#### Software

Dionysus software [102] with CGAL library [103] is used for persistent homology computation on alpha complex. Javaplex [104] and Dipha [43] software packages are used for persistent homology computation on Vietoris-Rips complex. The neural networks are realized using Keras [105] wrapper of Theano [106] backend. Various functions from Numpy and Scipy [107] packages are used to process data and evaluate the performance.

## Supporting information

S1 Text Handcrafted features. Handcrafted auxiliary features for prediction of protein folding free energy change upon mutation.
(PDF)

S2 Text Network architectures. Detailed architectures and parameters of the neural networks introduced in this work.
(PDF)

S1 Code Binding topological features. Source code for the generation of 1D image-like topological features for the binding problem.
(ZIP)

S2 Code Mutation topological features. Source code for the generation of 1D image-like topological features for the mutation problem.
(ZIP)
