# Supplementary material for: TopologyNet: Topology based deep convolutional and multi-task neural networks for biomolecular property predictions
Source: PLoS Comput Biol. 2017 Jul 27;13(7):e1005690. doi: 10.1371/journal.pcbi.1005690 (PMC5549771; doi:10.1371/journal.pcbi.1005690)
Supplement: S1 Text — Handcrafted auxiliary features for prediction of protein folding free energy change upon mutation. (PDF) [file pcbi.1005690.s001.pdf]

TopologyNet: Topology based deep convolutional and multi-task neural networks for biomolecular property predictions — Handcrafted auxiliary features for the mutation example

Zixuan Cang and Guowei Wei

For the prediction of protein folding free energy change upon mutation, there are factors that are crucial to protein stability but can not be easily captured by persistent homology analysis, such as electrostatics. Also, there exists valuable sequential data base that can further enhance the performance of the predictor. Therefore, handcrafted features regarding the aforementioned aspects are generated and described in details in this document.

The atomic features are computed for each atom or each atom pair and are then summed over according to their element types and their locations. We consider the following atom types, $e_1 : \mathrm{C}, e_2 : \mathrm{N}, e_3 : \mathrm{O}, e_4 : \mathrm{S}, e_5 : \mathrm{H}, e_6 : \{\mathrm{C}, \mathrm{N}, \mathrm{O}, \mathrm{S}\}$, and $e_7 : \{\mathrm{C}, \mathrm{N}, \mathrm{O}, \mathrm{S}, \mathrm{H}\}$, and the following locations, l_1 : at the mutation site, l_2: within 10 Å of the mutation site but not at the mutation site, and l_3: with a distance greater than 10 Å to the mutation site while smaller than a cutoff of 40 Å. All features except the PSSM and the neighborhood amino acid composition are computed for both the mutant and the wild type. The difference of the features between the two structures are also taken into consideration.

Surface area Solvent excluded surface area for each atom is computed. A total of 21 area features are generated for either the mutant or the wild type by summing over combinations of 7 element types $e_1 - e_7$ and 3 locations $l_1 - l_3$. The difference between the mutant and the wild type gives rise to other 21 features. Therefore, there are 63 surface area features. The atomic surface area is computed with our in-house software package ESES.[5] ESES is available at `http://weilab.math.msu.edu/ESES/`.

van der Waals interaction The van der Waals interaction is modeled by the Lennard-Johns potential and is computed on each heavy atom by contrasting the atom against all other heavy atoms. A total of 63 features are generated by summing over the combinations of element types e_1, e_2, e_3, e_4, e_6 and locations $l_1 - l_3$.

Partial charge The atomic partial charges approximate the electron density. A total of 63 charge features are generated by summing over the combinations of element types $e_1 - e_7$ and locations $l_1 - l_3$. Another set of 63 charge features are generated by taking the absolute value of the partial charge on each atom before summations. The atomic partial charges are assigned using PDB2PQR software package.[1] An example usage of the software is "`pdb2pqr -ff=amber -ph-calc-method=propka -with-ph=7.0 input.pdb output.pqr`". This will also generate the file that describes the pKa information.

Coulomb interaction The Coulomb interaction energy is computed on each atom against all other atoms. Sixty three features are generated by summing over combinations of element types e_1, e_2, e_3, e_4, e_6 and locations $l_1 - l_3$. Another set of 63 Coulomb interaction features are obtained by taking the absolute value on each atom before summations.

Atomic electrostatic solvation free energy Atomic electrostatic solvation free energy for each atom is modeled with the Poisson Boltzmann model. Sixty three features are generated by summing over the combinations of element types $e_1 - e_7$ and locations $l_1 - l_3$. The atomic electrostatic solvation free energy is modeled with Poisson Boltzmann equation solved using our in-house software MIBPB. The MIBPB software can be found at `http://weilab.math.msu.edu/MIBPB/`. An example usage is "`mibpb $pdbid h=0.5`", which will carry out the Poisson Boltzmann computation for the input file `$pdbid.pqr` with grid size 0.5. Gird size of 0.5 Å is recommended while 0.8 Å is also acceptable.

The construction of the atomic features described above is further described in Algorithm 1.

Neighborhood amino acid composition Amino acid residues are grouped into hydrophobic, polar, positively charged, negatively charged, and special cases. The residues within 10 Å of the mutation site are examined. The count and percentage of nearby residues in each group are used as features describing the environment

of the mutation site providing 10 features. The accumulation, average, and variance of volume, surface area, weight, and hydropathy scores of the nearby residues are also calculated providing another 12 features.

pKa shifts The pKa values of ionizable groups are predicted. The maximum and summation of absolute value of pKa changes and summation of pKa changes between the wild type and the mutant as well as the largest pKa changes in both positive and negative directions are calculated resulting in 5 features. The pKa values and the differences between the wild type and the mutant at the mutation site, C terminal, and N terminal are also calculated resulting in another 9 features. Finally, the accumulated absolute and net pKa shifts for each of the seven ionizable groups make up an extra of 14 features. The pKa values of the ionizable groups are computed using PROPKA software package.[4]

Secondary structure The probability scores for the mutation site to be in a coil, helix, and strand as well as the torsion angle are predicted from the amino acid sequences for the wild type and the mutant. The secondary structure of the mutation site are also directly identified from the protein structure. The difference between the wild type and the mutant are also included resulting in 18 features. The secondary structure probability scores are predicted using SPIDER software package.[2] An example usage of SPIDER software is `"SPIDER2_local/misc/pred_pssm.py $PSSMFile"`

Position-specific scoring matrix (PSSM) The conservation score in the position-specific scoring matrix of the mutation site to be the amino acid in the wild type and the mutant as well as the difference of the two are used as features. Specifically, we collect the two PSSM scores for the mutation site to be in the wild residue type and mutant residue type and the difference between the two from the PSSMs constructed from wild type sequences. The PSSMs are computed using the psiblast command from BLAST+ software suite.[3] The specific version used is BLAST+/2.2.31 and an example usage is `"psiblast -query -db $PathToNRDatabase -num_iterations 5 -out $OutFileName -out_ascii_pssm $PSSMFileName"`. If the query sequence is too short and there is no hit found, the option `-evalue` can be set to a bigger number to loosen the criteria of including an entry as hit.

Algorithm 1 The detailed steps of constructing atomic features (solvation energy, surface ares, partial charge, van der Waals, Coulomb).

```
function DistanceToMutationSite(atom)
    curdis ← ∞
    for i = 1 : m do                              ▷ Loop over all m atoms at the mutation site
        dis ← Euclidean(atom, atom_i)             ▷ The distance between two atoms
        if dis < curdis then
            curdis ← dis
        end if
    end for
    return dis
end function

function AtomLocation(atom)
    if atom is at mutation site then
        return 1
    else if DistanceToMutationSite(atom) < 10 Å then
        return 2
    else
        return 3
    end if
end function

function ElementGroup(groupindex)
    if groupindex==1 then
        return {C}
    else if groupindex==2 then
        return {N}
    else if groupindex==3 then
        return {O}
```

```
    else if groupindex==4 then
        return {S}
    else if groupindex==5 then
        return {H}
    else if groupindex==6 then
        return {C, N, O, S}
    else if groupindex==7 then
        return {C, N, O, S, H}
    end if
end function

function CONSTRUCTATOMICFEATURE(protein)
    featureatomic ← Empty vector
    featuresolveng ← Zeros_{3×7}
    for i = 1 : n do                                    ▷ Loop over the n atoms of the protein
        for ie = 1 : 7 do
            if ELEMENT(atom_i) in ELEMENTGROUP(ie) then
                featuresolveng[ATOMLOCATION(atom),ie] += SOLVENG(atom_i)
            end if
        end for
    end for
    Append FLATTEN(featuresolveng) to featureatomic
    featurearea ← Zeros_{3×7}
    for i = 1 : n do
        for ie = 1 : 7 do
            if ELEMENT(atom_i) in ELEMENTGROUP(ie) then
                featurearea[ATOMLOCATION(atom),ie] += SUFACEAREA(atom_i)
            end if
        end for
    end for
    Append FLATTEN(featurearea) to featureatomic
    featurecharge ← Zeros_{3×7×2}
    for i = 1 : n do
        for ie = 1 : 7 do
            if ELEMENT(atom_i) in ELEMENTGROUP(ie) then
                featurecharge[ATOMLOCATION(atom),ie,1] += PARTIALCHARGE(atom_i)
                featurecharge[ATOMLOCATION(atom),ie,2] += Abs(PARTIALCHARGE(atom_i))
            end if
        end for
    end for
    Append FLATTEN(featurecharge) to featureatomic
    featurevdw ← Zeros_{3×5}
    for i = 1 : n do
        for j = 1 : n do
            for ie in {1, 2, 3, 4, 6} do
                if ELEMENT(atom_i) in ELEMENTGROUP(ie) and ELEMENT(atom_j) in ELEMENTGROUP(6) then
                    featurevdw[ATOMLOCATION(atom),ie] += VANDERWAALS(atom_i, atom_j)
                end if
            end for
        end for
    end for
    Append FLATTEN(featurevdw) to featureatomic
    featureclb ← Zeros_{3×5×2}
    for i = 1 : n do
```

```
        for j = 1 : n do
            for ie in {1, 2, 3, 4, 6} do
                if Element(atom_i) in ElementGroup(ie) and Element(atom_j) in ElementGroup(7) then
                    featurevdw[AtomLocation(atom),ie,1] += Coulomb(atom_i, atom_j)
                    featurevdw[AtomLocation(atom),ie,2] += Abs(Coulomb(atom_i, atom_j))
                end if
            end for
        end for
    end for
    Append Flatten(featureclb) to featureatomic
    return featureatomic
end function
```

References

[1] T. J. Dolinsky, P. Czodrowski, H. Li, J. E. Nielsen, J. H. Jensen, G. Klebe, and N. A. Baker. PDB2PQR: expanding and upgrading automated preparation of biomolecular structures for molecular simulations. *Nucleic Acids Research*, 35(S2):W522–5, 2007.

[2] Rhys Heffernan, Kuldip Paliwal, James Lyons, Abdollah Dehzangi, Alok Sharma, Jihua Wang, Abdul Sattar, Yuedong Yang, and Yaoqi Zhou. Improving prediction of secondary structure, local backbone angles, and solvent accessible surface area of proteins by iterative deep learning. *Scientific reports*, 5, 2015.

[3] Mark Johnson, Irena Zaretskaya, Yan Raytselis, Yuri Merezhuk, Scott McGinnis, and Thomas L Madden. Ncbi blast: a better web interface. *Nucleic Acids Research*, 36(suppl 2):W5–W9, 2008.

[4] H. Li, A. D. Robertson, and J. H. Jensen. Very fast empirical prediction and rationalization of protein pka values. *Proteins*, 61(4):704–21, 2005.

[5] Beibei Liu, Bao Wang, Rundong Zhao, Yiying Tong, and Guo Wei Wei. ESES: software for Eulerian solvent excluded surface. *Journal of Computational Chemistry*, 38:446–466, 2017.
