# Supplementary material for: TopologyNet: Topology based deep convolutional and multi-task neural networks for biomolecular property predictions
Source: PLoS Comput Biol. 2017 Jul 27;13(7):e1005690. doi: 10.1371/journal.pcbi.1005690 (PMC5549771; doi:10.1371/journal.pcbi.1005690)
Supplement: S2 Text — Detailed architectures and parameters of the neural networks introduced in this work. (PDF) [file pcbi.1005690.s002.pdf]

Architecture of TNet-BP

Optimizer: Adam, learning rate=0.0001. **Mini-batch size**: 16. **Epoch number**: 2000. **Weight initialize**: glorot_uniform. **Notations**: Conv1D(filter number, filter size, activation, weight initialization), Dense(neuron number, activation, weight initialization), Dropout(dropout rate), Pooling(pooling size). The input is generated following the source code in S1 Code.

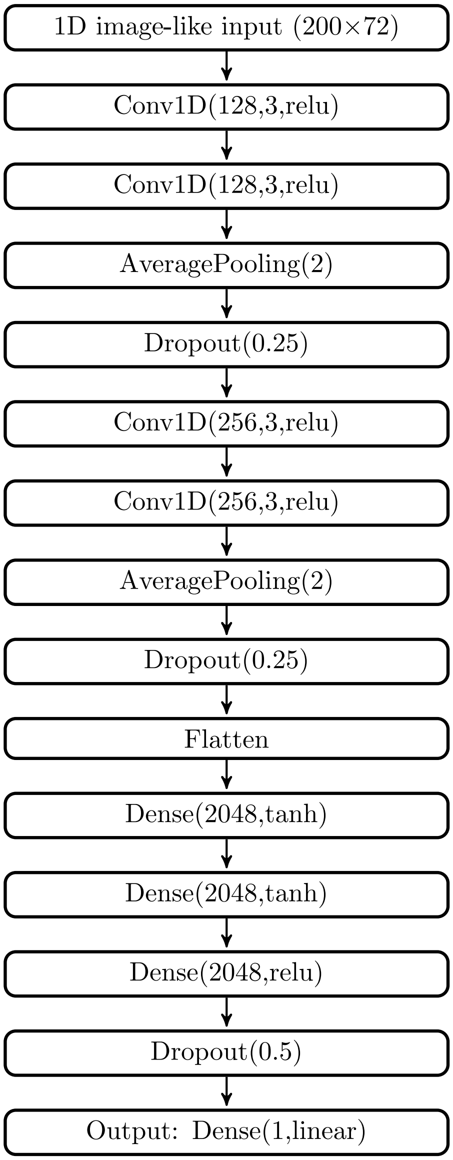

Architectures of TNet-MP-1 (only topological input without the dashed part) and TNet-MP-2 (topological input and auxiliary features with the dashed part)

Optimizer: Adam, learning rate=0.0001. **Mini-batch size**: 16. **Epoch number**: 500. **Notations**: Conv1D(filter number, filter size, activation, weight initialization), Dense(neuron number, activation, weight initialization), Dropout(dropout rate), Pooling(pooling size). The 1D image-like input is generated following the source code in S2 Code and the handcrafted feature input is generated according to the description and pseudocode in S1 Text.

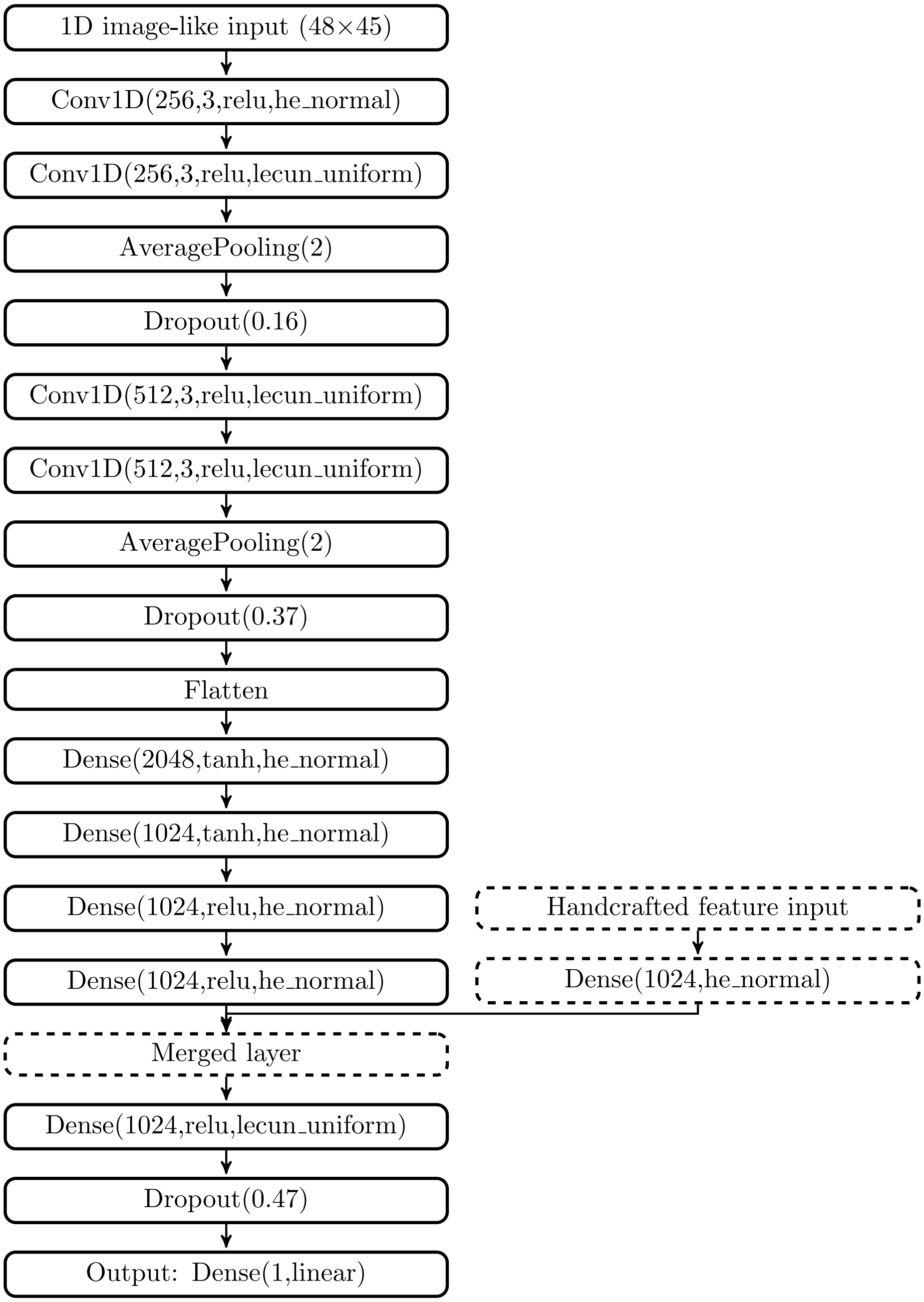

Architectures of TNet-MMP-1 (single-task without the dashed part) and TNet-MMP-2 (multitask with the dashed part)

Optimizer: Adam, learning rate=0.0001. **Mini-batch size**: 16. **Training**: 500 epoch (TNet-MMP-1); 800 epoch on globular branch followed by 100 epoch on membrane branch (TNet-MMP-2). **Notations**: Conv1D(filter number, filter size, activation, weight initialization), Dense(neuron number, activation, weight initialization), Dropout(dropout rate), Pooling(pooling size). The input is generated following the source code in S2 Code.

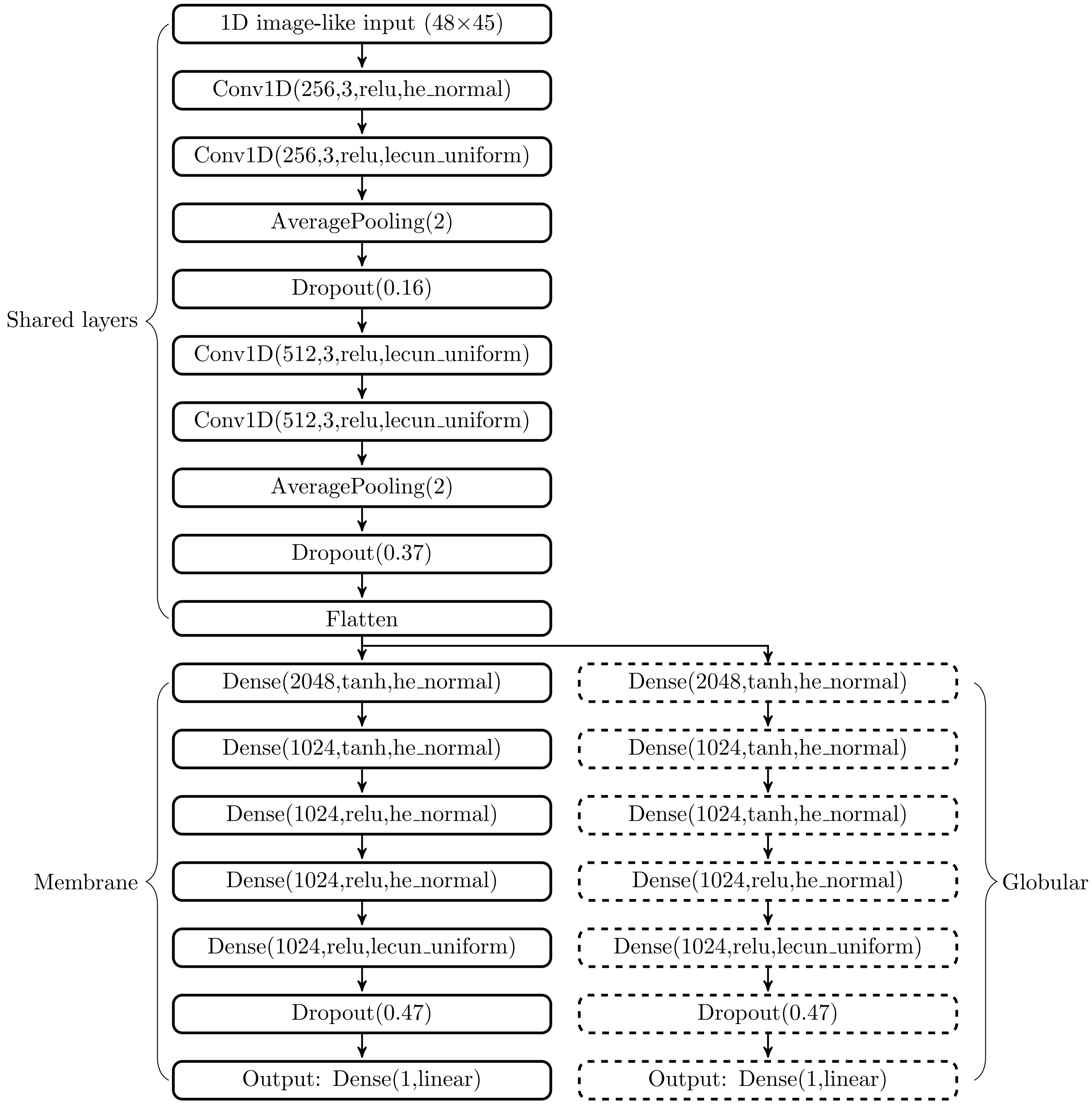

Keras code for TNet-BP (Keras==1.1.2)

```
import numpy as np
from keras.models import Sequential
from keras.layers import Dense, Dropout, Activation, Flatten
from keras.optimizers import SGD, Adam
from keras.layers import Convolution1D, MaxPooling1D, AveragePooling1D

# Load X_train, X_test, Y_train, Y_test as feature inputs and labels

model = Sequential()
model.add(Convolution1D(128, 3, border_mode='same', input_shape=X_train.shape[1:]))
model.add(Activation('relu'))
model.add(Convolution1D(128, 3))
model.add(Activation('relu'))
model.add(AveragePooling1D(pool_length=2, stride=None, border_mode='valid'))
model.add(Dropout(0.25))
model.add(Convolution1D(256, 3, border_mode='same'))
model.add(Activation('relu'))
model.add(Convolution1D(256, 3))
model.add(Activation('relu'))
model.add(AveragePooling1D(pool_length=2, stride=None, border_mode='valid'))
model.add(Dropout(0.25))
model.add(Flatten())
model.add(Dense(2048))
model.add(Activation('tanh'))
model.add(Dense(2048))
model.add(Activation('tanh'))
model.add(Dense(2048))
model.add(Activation('relu'))
model.add(Dropout(0.5))
model.add(Dense(1))
model.add(Activation('linear'))
adam = Adam(lr=0.0001)
model.compile(loss='mean_squared_error',
              optimizer=adam,
              metrics=['mean_squared_error'])
batch_size = 16
nb_epoch = 2000
history = model.fit(X_train, Y_train,
          batch_size=batch_size,
          nb_epoch=nb_epoch,
          validation_data=(X_test, Y_test),
          shuffle=True)
```

Keras code for TNet-MP-1 (Keras==1.1.2)

```
import numpy as np
from keras.models import Sequential
from keras.layers import Dense, Dropout, Activation, Flatten, Merge
from keras.optimizers import SGD, Adam
from keras.layers import Convolution1D, MaxPooling1D, AveragePooling1D

# X_train and X_test are the image like features from persistent homology.
# Y_train and Y_test are labels.
# X_cl_train and X_cl_test are the auxiliary features.

model = Sequential()
model.add(Convolution1D(256, 3, border_mode='same', input_shape=X_train.shape[1:],
          init='he_normal'))
model.add(Activation('relu'))
model.add(Convolution1D(256, 3, init='lecun_uniform'))
model.add(Activation('relu'))
model.add(AveragePooling1D(pool_length=2, stride=None, border_mode='valid'))
model.add(Dropout(0.16))
model.add(Convolution1D(512, 3, border_mode='same', init='lecun_uniform'))
model.add(Activation('relu'))
model.add(Convolution1D(512, 3, init='lecun_uniform'))
model.add(Activation('relu'))
model.add(AveragePooling1D(pool_length=2, stride=None, border_mode='valid'))
model.add(Dropout(0.37))
model.add(Flatten())
model_cl = Sequential()
model_cl.add(Dense(1024, input_dim=X_cl_train.shape[1], init='he_normal'))
model.add(Dense(2048, init='he_normal'))
model.add(Activation('tanh'))
model.add(Dense(1024, init='he_normal'))
model.add(Activation('tanh'))
model.add(Dense(1024, init='he_normal'))
model.add(Activation('relu'))
model.add(Dense(1024, init='he_normal'))
model.add(Activation('relu'))
model_merged = Sequential()
model_merged.add(Merge([model, model_cl], mode = 'concat'))
model_merged.add(Dense(1024, init='lecun_uniform'))
model_merged.add(Activation('relu'))
model_merged.add(Dropout(0.47))
model_merged.add(Dense(1))
model_merged.add(Activation('linear'))
adam = Adam(lr=0.0001)
model_merged.compile(loss='mean_squared_error',
                     optimizer=adam,
                     metrics=['mean_squared_error'])
batch_size = 16
```

```
nb_epoch = 500
history = model_merged.fit([X_train, X_cl_train], Y_train,
          batch_size=batch_size,
          nb_epoch=nb_epoch,
          validation_data=([X_test, X_cl_test], Y_test),
          shuffle=True)
```

Keras code for TNet-MMP-1 (Keras==1.1.2)

```
import numpy as np
from keras.models import Model, Sequential
from keras.layers import Input, Dense, Dropout, Flatten
from keras.optimizers import SGD, Adam
from keras.layers import Convolution1D, MaxPooling1D, AveragePooling1D

# X_S2648, Y_S2648 are feature and label for the globular protein dataset
# X_M223_train, X_M223_test, Y_M223_train, Y_M223_test
# are the features and labels for the membrane protein dataset

inputs = Input(shape=X_M223_train.shape[1:])
model_shared = Convolution1D(256, 3, border_mode='same',
                 activation='relu', init='he_normal')(inputs)
model_shared = Convolution1D(256, 3, activation='relu',
                 init='lecun_uniform')(model_shared)
model_shared = AveragePooling1D(pool_length=2, stride=None,
                 border_mode='valid')(model_shared)
model_shared = Dropout(0.16)(model_shared)
model_shared = Convolution1D(512, 3, border_mode='same',
                 activation='relu', init='lecun_uniform')(model_shared)
model_shared = Convolution1D(512, 3, activation='relu',
                 init='lecun_uniform')(model_shared)
model_shared = AveragePooling1D(pool_length=2, stride=None,
                 border_mode='valid')(model_shared)
model_shared = Dropout(0.37)(model_shared)
model_S2648 = Flatten()(model_shared)
model_S2648 = Dense(2048, activation='tanh', init='he_normal')(model_S2648)
model_S2648 = Dense(1024, activation='tanh', init='he_normal')(model_S2648)
model_S2648 = Dense(1024, activation='relu', init='he_normal')(model_S2648)
model_S2648 = Dense(1024, activation='relu', init='he_normal')(model_S2648)
model_S2648 = Dense(1024, activation='relu', init='lecun_uniform')(model_S2648)
model_S2648 = Dropout(0.47)(model_S2648)
model_S2648 = Dense(1, activation='linear')(model_S2648)
model_M223 = Flatten()(model_shared)
model_M223 = Dense(2048, activation='tanh', init='he_normal')(model_M223)
model_M223 = Dense(1024, activation='tanh', init='he_normal')(model_M223)
model_M223 = Dense(1024, activation='relu', init='he_normal')(model_M223)
model_M223 = Dense(1024, activation='relu', init='he_normal')(model_M223)
model_M223 = Dense(1024, activation='relu', init='lecun_uniform')(model_M223)
model_M223 = Dropout(0.47)(model_M223)
model_M223 = Dense(1, activation='linear')(model_M223)
ModelM223 = Model(input=inputs, output=model_M223)
ModelS2648 = Model(input=inputs, output=model_S2648)
adam = Adam(lr=0.0001)
ModelM223.compile(optimizer=adam, loss='mean_squared_error',
                  metrics=['mean_squared_error'])
ModelS2648.compile(optimizer=adam, loss='mean_squared_error',
```

```
                               metrics=['mean_squared_error'])
ModelS2648.fit(X_S2648, Y_S2648, batch_size=16, nb_epoch=800, shuffle=True)
ModelM223.fit(X_M223_train, Y_M223_train, batch_size=16, nb_epoch=100,
              validation_data=(X_M223_test, Y_M223_test), shuffle=True)
```
